# Integrating Clinical Classifications Software Refined, Process Indicators, and Geographic Information System Mapping to Inform Population Health Management: Development of an Interactive Dashboard

**DOI:** 10.2196/80431

**Published:** 2026-07-15

**Authors:** Joshua Kuan Tan, Hao Yi Tan, Gerald Gui Ren Sng, Sing Yi Chia, Su-Yen Goh, Julian Thumboo, Yong Mong Bee

**Affiliations:** 1 Health Services Research Unit, Singapore General Hospital, Outram Road, Singapore, 169608, Singapore, 65 62223322; 2 Department of Endocrinology, Singapore General Hospital, Singapore, Singapore; 3 Future Health Systems Department, Singapore General Hospital, Singapore, Singapore

**Keywords:** population health management, population health analytics, health informatics, geographic information systems, data visualization, diabetes mellitus, complications

## Abstract

**Background:**

Population health management requires tools that transform complex clinical data into actionable insights to guide care coordination, community outreach, and system-level planning.

**Objective:**

The objective of this study is to develop and apply a population health intelligence dashboard that integrates inpatient utilization, process indicators, and health status data for patients with diabetes mellitus, using a clinically meaningful classification system and geospatial visualization.

**Methods:**

We used data from the SingHealth Diabetes Registry (SDR; 2019‐2024) to build an interactive dashboard using R Shiny (Posit Software). A semiautomated mapping algorithm was developed to map *ICD-10-AM* (*International Classification of Diseases, 10th Revision, Australian Modification*) principal diagnosis codes into CCSR (Clinical Classifications Software Refined) categories. We built an interactive dashboard in R Shiny incorporating 3 analytic domains: inpatient utilization (by admission count, length of stay, and prolonged stays), diabetes care process indicators, and health status indicators (eg, comorbidities, laboratory results, and diabetes-related complications). Geographic information system mapping enabled spatial visualization by patients’ residential locations.

**Results:**

Diabetes mellitus with complication (END003) was the leading cause of admission (7.0%‐8.1% annually), followed by pneumonia (RSP002, 3.7%‐5.1%), fluid and electrolyte disorders (END011, 3.4%‐4.1%), and skin infections (SKN001, 2.8%‐3.1%). In 2024, top *ICD-10-AM* diagnoses under END003 included E1122—type 2 diabetes mellitus with established diabetic nephropathy, E1173—type 2 diabetes mellitus with foot ulcer due to multiple causes, and E1172—type 2 diabetes mellitus with features of insulin resistance. For END011, the most frequent diagnosis codes were E877—fluid overload, R18—ascites, E875—hyperkalemia, and hypo-osmolality and hyponatremia. In total, SingHealth Diabetes Registry patients accounted for 687,062 inpatient bed days in 2024. Circulatory conditions (eg, cerebral infarction and heart failure) contributed 124,417 (17.7%) bed days, while injuries (eg, hip fractures and surgical complications) accounted for 86,541 (12.6%) bed days. CCSR-based analyses revealed distinct patterns when comparing conditions driving admission frequency versus prolonged length of stay. GIS mapping identified residential clusters with high inpatient utilization, unmet care processes, and poor cardiometabolic control, supporting region-specific intervention planning.

**Conclusions:**

The dashboard demonstrates a novel, interactive approach to visualizing inpatient utilization, care gaps, and health status, enabling targeted, place-based interventions. It represents a scalable framework for operationalizing population health intelligence across other chronic disease areas and health care systems.

## Introduction

Across the globe, many countries are undergoing demographic and epidemiological transitions, marked by a rising burden of chronic conditions such as diabetes, hypertension, and hyperlipidemia, along with their associated complications. These conditions significantly increase health care utilization and place growing strain on health systems and resources. Population health management (PHM) has emerged as a strategic approach to address these challenges by proactively identifying at-risk populations, coordinating care across settings, and implementing targeted interventions to prevent complications, reduce avoidable utilization, and improve long-term health outcomes [1].

PHM emphasizes addressing the broader determinants of health for populations within defined geographic areas, often in collaboration with a range of health and community partners [2]. A critical enabler of PHM is the availability of robust information technology infrastructure that can integrate diverse data sources to support in-depth analyses, inform targeted interventions, and assess the outcomes of these interventions [1,2].

Three key challenges commonly affect PHM: identifying the clinical drivers of health care utilization across a health system, making sense of complex disease and utilization data in a structured, actionable way, and leveraging digital technologies to design programs that reduce preventable utilization. Addressing these challenges requires integrated, multifunctional data dashboards and analytic tools [1].

A recent scoping review found that most dashboards served either clinical purposes (direct care, PHM, and care coordination) or administrative purposes (performance monitoring, utilization tracking, and resource management), and few served both purposes [3]. Furthermore, clinical dashboards often had a narrow disease focus, typically targeting specific conditions or objectives. Notably, the review found no dashboard that integrated PHM, care coordination, and utilization tracking—functions critical to delivering integrated, proactive care.

In this paper, we describe the development of a population health intelligence dashboard designed to uncover both clinical and administrative priorities for patients with diabetes mellitus. The dashboard was engineered to dynamically support varying levels of disease specificity—enabling users to explore health care utilization trends across a broad range of conditions or drill down into specific disease areas as needed. By integrating core functions such as PHM, care coordination, and utilization tracking, the platform facilitates data-driven planning and supports the design of targeted interventions to improve system-level performance and public health outcomes.

## Methods

### Study Setting and Data Sources

We used data from the SingHealth Diabetes Registry (SDR), a comprehensive, multi-institutional repository of diabetes-related patient data from the Singapore Health Services (SingHealth) Regional Health System. SingHealth is the largest of the 3 public health care clusters in Singapore, comprising 4 acute hospitals, 5 national specialty centers, 3 community hospitals, and a network of 10 primary care polyclinics, and is responsible for the care of almost half of Singapore’s population.

The SDR integrates data from electronic medical records and clinical databases and includes all individuals aged 18 years and older diagnosed with diabetes mellitus, excluding those with prediabetes. Cases are identified annually using a combination of diagnostic criteria, including *International Classification of Diseases*, *9th and 10th Revisions* (*ICD-9* and *ICD-10*), prescription records, and laboratory test results [4]. We included patients recorded in the SDR during the 5-year study period from January 1, 2019, to December 31, 2024.

The SDR was selected as the data source for this study because it is the most established and quality-assured chronic disease registry in our institution. It offers a high degree of data completeness, standardized variable definitions, and regular validation processes, making it well-suited for longitudinal and system-wide analysis. Its integration across care settings—from primary care to tertiary hospitals—enables a holistic view of population health among individuals living with a chronic condition of public health importance.

### Key Dashboard Features

The development of the dashboard was iterative and guided by stakeholder engagement to address both clinical and administrative priorities. Through this process, 3 core analytic domains consistently emerged as critical for population health decision-makers and clinicians managing diabetes care: (1) health status indicators, (2) process indicators, and (3) inpatient utilization—detailed in Textbox 1.

Textbox 1. **Variables used for analytical domains.**

**Health status indicators**

- Demographic filters
  - Gender
  - Age band (<40 y, 40-59 y, 60-69 y, 70-79 y, >80 y)

- Health and comorbidity filters
  - Ethnicity
  - Asian BMI category
  - Housing type
  - Smoking status
  - Diabetes type
  - Average hemoglobin A_1c_ (HbA_1c_) (%) in the calendar year
  - Hypertension status
  - Hyperlipidemia status
  - Average low-density lipoprotein cholesterol in calendar year
  - Estimated glomerular filtration rate (eGFR) category based on average eGFR in calendar year
  - Dialysis status

- Diabetes-related complications
  - Ischemic heart disease status
  - Acute myocardial infarction status
  - Peripheral arterial disease status
  - Cerebrovascular accident (ischemic and hemorrhagic) status

**Process indicators (in calendar year)**

- No low-density lipoprotein cholesterol
- Less than 2 HbA_1c_
- No diabetic retinal photography
- No diabetic retinal photography or ophthalmology visit
- No eGFR lab result
- No urine albumin creatinine ratio lab result
- No diabetic foot screening

Health status indicators captured markers of disease control, concomitant comorbidities, and prevalent diabetes-related complications. The methodology for identifying diabetes-related complications has been previously described [5]. Process indicators reflect adherence to local evidence-based care protocols [6] and provide insight into the consistency and quality of chronic disease management across the care continuum. Inpatient utilization represents downstream, resource-intensive events that may indicate priority areas for disease control or access to care, offering actionable insight into clinical drivers and the geographic distribution of hospital admissions.

Together, these domains form the conceptual foundation of the dashboard, informing its data architecture, user interface (UI), and decision-support functions. By integrating clinical outcomes, care quality metrics, and utilization data, the dashboard provides a comprehensive and actionable view of diabetes care—bridging community-based and hospital-level programs while informing targeted interventions and policy decisions.

### Assessing Inpatient Utilization Using the Clinical Classifications Software Refined

To facilitate interpretation of the highly granular and diverse clinical conditions represented in *ICD-10*, we utilized the CCSR (Clinical Classifications Software Refined), a software tool developed by the US Agency for Healthcare Research and Quality [7]. The CCSR groups *ICD-10-CM* (*International Classification of Diseases, 10th Revision, Clinical Modification*) codes into approximately 530 clinically meaningful categories, enabling research about disease-specific health care utilization and outcomes [7,8].

As our health care system uses the *ICD-10-AM* (*International Classification of Diseases, 10th Revision, Australian Modification*), we developed a semiautomated algorithm for mapping *ICD-10-AM* codes to corresponding CCSR categories, adapting the methodology described by Malecki et al [8]. The algorithm is based on the official CCSR tool (v2025.1; United States Agency for Healthcare Research and Quality) [7], which assigns a default CCSR code to each principal diagnostic code. The matching algorithm was developed and implemented in R (version 4.4.3; R Core Team), and the code is available in Box 1 in Multimedia Appendix 1.

Unique *ICD-10-AM* principal diagnostic codes were extracted from the SDR and mapped according to the schematic shown in Figure S1 in Multimedia Appendix 1. Codes with exact matches to *ICD-10-CM* entries in the official CCSR file were assigned the default CCSR category (direct mapping). Codes without a direct match underwent an iterative mapping process using the hierarchical structure of *ICD-10*, progressively matching to parent, child, or sibling codes assumed to share similar clinical characteristics.

Mapping proceeded sequentially—beginning with 5-character *ICD-10-CM* codes, followed by 4-character and 3-character codes—allowing for both one-to-one and one-to-many mappings. Unmatched codes were truncated as needed to enable parent or grandparent-level matching. This process was repeated iteratively until all possible matches were exhausted.

Finally, 2 clinicians (authors JKT and HYT) independently reviewed all mapped codes. Those mapped to a single CCSR category were validated directly, while codes mapped to multiple categories or those that remained unmatched were manually reviewed and assigned to the most appropriate category, using the official CCSR documentation as reference. Discrepancies were resolved through consensus discussion, and where needed, a third clinician (the author GGRS) was consulted.

### Data Visualization

To visualize the data across the 3 analytic domains, we developed an interactive web-based application (herein referred to as the dashboard) using Shiny (Posit Software), an R package to build interactive UIs. For inpatient utilization, we implemented interactive nested treemaps to display the hierarchical relationships between CCSR categories and *ICD-10-AM* codes. These treemaps allowed users to easily identify high-volume classifications and to apply filters by year, health care institution, clinical specialty, and length of stay (LOS).

Geographic information system (GIS) mapping was used to visualize spatial patterns in inpatient utilization, health status indicators, and process indicators, supporting place-based planning and targeted community interventions. Additional geographic markers for community health care assets (eg, general practitioner [GP] clinics, polyclinics, active aging centers [AACs], and other community partners) were also displayed. Geocoding was performed using the Singapore Land Authority’s OneMap application programming interface [9].

The dashboard was developed using a collaborative and iterative approach, incorporating feedback from clinicians and health system stakeholders through cyclical testing and refinement [10,11]. It was designed to be code-free and intuitive, allowing users to customize data views via interactive filters and toggles, with key metrics surfaced rapidly through pop-up windows and dynamic counter boxes, powered by reactive UI elements and real-time data binding to support efficient, insight-driven exploration.

### Ethical Considerations

The SingHealth Centralised Institutional Review Board determined that an ethics review was not required for the use of deidentified data obtained during routine clinical care (SingHealth Centralised Institutional Review Board reference number 2022/2133). As all participant data were deidentified, a waiver for participant consent was also obtained.

## Results

### Inpatient Utilization Within the SDR

Between 2019 and 2024, there were 458,155 inpatient admission events in the SDR across the SingHealth cluster (Table 1). These events involved 5892 unique primary discharge diagnoses coded using *ICD-10-AM*. Using the *ICD-10-CM*–based CCSR framework, these codes were algorithmically mapped to CCSR categories, as illustrated in Figure S2 in Multimedia Appendix 1.

**Table 1.** Top 5 most frequent clinical conditions (Clinical Classification Software Refined [CCSR] categories) for inpatient admissions (2019‐2024).

Year: number of inpatient admissions and top 5 CCSR categories (code)	Values, n (%)
Year 2019: 71,615 admissions	
Diabetes mellitus with complications (END003)	5792 (8.1)
Pneumonia (except that caused by tuberculosis) (RSP002)	2635 (3.7)
Fluid and electrolyte disorders (END011)	2284 (3.2)
Coronary atherosclerosis and other heart diseases (CIR011)	2261 (3.2)
Urinary tract infections (GEN004)	2215 (3.1)
Year 2020: 69,735 admissions	
Diabetes mellitus with complications (END003)	5515 (7.9)
Fluid and electrolyte disorders (END011)	2869 (4.1)
Pneumonia (except that caused by tuberculosis) (RSP002)	2607 (3.7)
Urinary tract infections (GEN004)	2269 (3.3)
Skin and subcutaneous tissue infections (SKN001)	2198 (3.2)
Year 2021: 71,106 admissions	
Diabetes mellitus with complications (END003)	6111 (8.6)
Pneumonia (except that caused by tuberculosis) (RSP002)	2867 (4.0)
Fluid and electrolyte disorders (END011)	2481 (3.5)
Urinary tract infections (GEN004)	2268 (3.2)
Skin and subcutaneous tissue infections (SKN001)	2164 (3.0)
Year 2022: 76,656 admissions	
Diabetes mellitus with complications (END003)	6204 (8.1)
Pneumonia (except that caused by tuberculosis) (RSP002)	3640 (4.7)
Fluid and electrolyte disorders (END011)	2618 (3.4)
Other specified upper respiratory infections (RSP006)	2358 (3.1)
Skin and subcutaneous tissue infections (SKN001)	2182 (2.8)
Year 2023: 82,308 admissions	
Diabetes mellitus with complications (END003)	6139 (7.5)
Pneumonia (except that caused by tuberculosis) (RSP002)	4237 (5.1)
Fluid and electrolyte disorders (END011)	2903 (3.5)
Skin and subcutaneous tissue infections (SKN001)	2466 (3.0)
Urinary tract infections (GEN004)	2322 (2.8)
Year 2024: 86,735 admissions	
Diabetes mellitus with complications (END003)	6078 (7.0)
Pneumonia (except that caused by tuberculosis) (RSP002)	4001 (4.6)
Fluid and electrolyte disorders (END011)	2988 (3.4)
Coronary atherosclerosis and other heart diseases (CIR011)	2984 (3.4)
Skin and subcutaneous tissue infections (SKN001)	2722 (3.1)

Briefly, of the 5892 *ICD-10-AM* codes, 5738 (97.4%) were successfully mapped, while 154 (2.6%) remained unmapped. Among the mapped codes, 4630 (80.7%) corresponded to a single CCSR category, whereas 1108 (19.3%) were linked to multiple categories. A comprehensive manual review referencing the original CCSR documentation was conducted to validate and refine the mappings; 1277 codes were manually modified or reassigned, and all *ICD-10-AM* codes were mapped to one of 422 CCSR categories.

### Top Clinical Conditions and Diagnoses Contributing to Admissions

Table 1 presents the 5 most frequent conditions by the CCSR category for inpatient admissions between 2019 and 2024. Across all years studied, diabetes mellitus with complication (END003) was the leading cause of admission, accounting for 7.0% to 8.1% of total hospitalizations annually. Pneumonia (RSP002) was the second most common condition in most years, contributing 3.7% to 5.1% of admissions. This was followed by fluid and electrolyte disorders (END011) at 3.4% to 4.1%, and skin and subcutaneous tissue infections (SKN001) at 2.8% to 3.1%.

Table 2 presents the 10 most frequent *ICD-10-AM* principal discharge codes under the CCSR categories “diabetes mellitus with complication” (END003) and “fluid and electrolyte disorders” (END011) for inpatient admissions in 2024.

**Table 2.** Top 10 most frequent *International Classification of Disease, 10th Revision, Australian Modification* (*ICD-10-AM*) principal discharge codes under the Clinical Classification Software Refined (CCSR) categories “diabetes mellitus with complication” (END003) and “fluid and electrolyte disorders” (END011) for inpatient admissions in 2024.

*ICD-10-AM* primary discharge codes	Values, n (%)
Top 10 codes in diabetes mellitus with complication (END003)	
E1122—type 2 diabetes mellitus with established diabetic nephropathy	1128 (18.6)
E1173—type 2 diabetes mellitus with foot ulcer due to multiple causes	874 (14.4)
E1172—type 2 diabetes mellitus with features of insulin resistance	468 (7.7)
E1129—type 2 diabetes mellitus with other specified kidney complications	467 (7.7)
E1164—type 2 diabetes mellitus with hypoglycemia	402 (6.6)
E1152—type 2 diabetes mellitus with peripheral angiopathy, with gangrene	273 (4.5)
E1422—unspecified diabetes mellitus with established diabetic nephropathy	217 (3.6)
E1464—unspecified diabetes mellitus with hypoglycemia	216 (3.6)
E1169—type 2 diabetes mellitus with other specified complication	206 (3.4)
E1165—type 2 diabetes mellitus with poor control	192 (3.2)
Top 10 codes in fluid and electrolyte disorders (END011)	
E877—fluid overload	1941 (65.0)
R18—ascites	290 (9.7)
E875—hyperkalemia	290 (9.7)
E871—hypo-osmolality and hyponatremia	217 (7.3)
E86—volume depletion	104 (3.5)
E876—hypokalemia	93 (3.1)
E872—acidosis	28 (0.9)
E870—hyperosmolality and hypernatremia	11 (0.4)
E878—other disorders of electrolyte and fluid balance, not elsewhere classified	9 (0.3)
E87—other disorders of fluid, electrolyte and acid-base balance	5 (0.2)

In 2024, within diabetes mellitus with complications (END003), the most common diagnoses were E1122—type 2 diabetes mellitus with established diabetic nephropathy (n=1128, 18.6%), E1173—type 2 diabetes mellitus with foot ulcer due to multiple causes (n=874, 14.4%), and E1172—type 2 diabetes mellitus with features of insulin resistance (n=468, 7.7%). Other frequently recorded conditions included hypoglycemia, peripheral angiopathy with gangrene, and poor glycemic control.

For fluid and electrolyte disorders (END011), the most frequent diagnosis codes were E877—fluid overload (n=1941, 65%), followed by R18—ascites (n=290, 9.7%), E875—hyperkalemia (n=290, 9.7%), and hypo-osmolality and hyponatremia (n=217, 7.3%).

**Figure 1.**
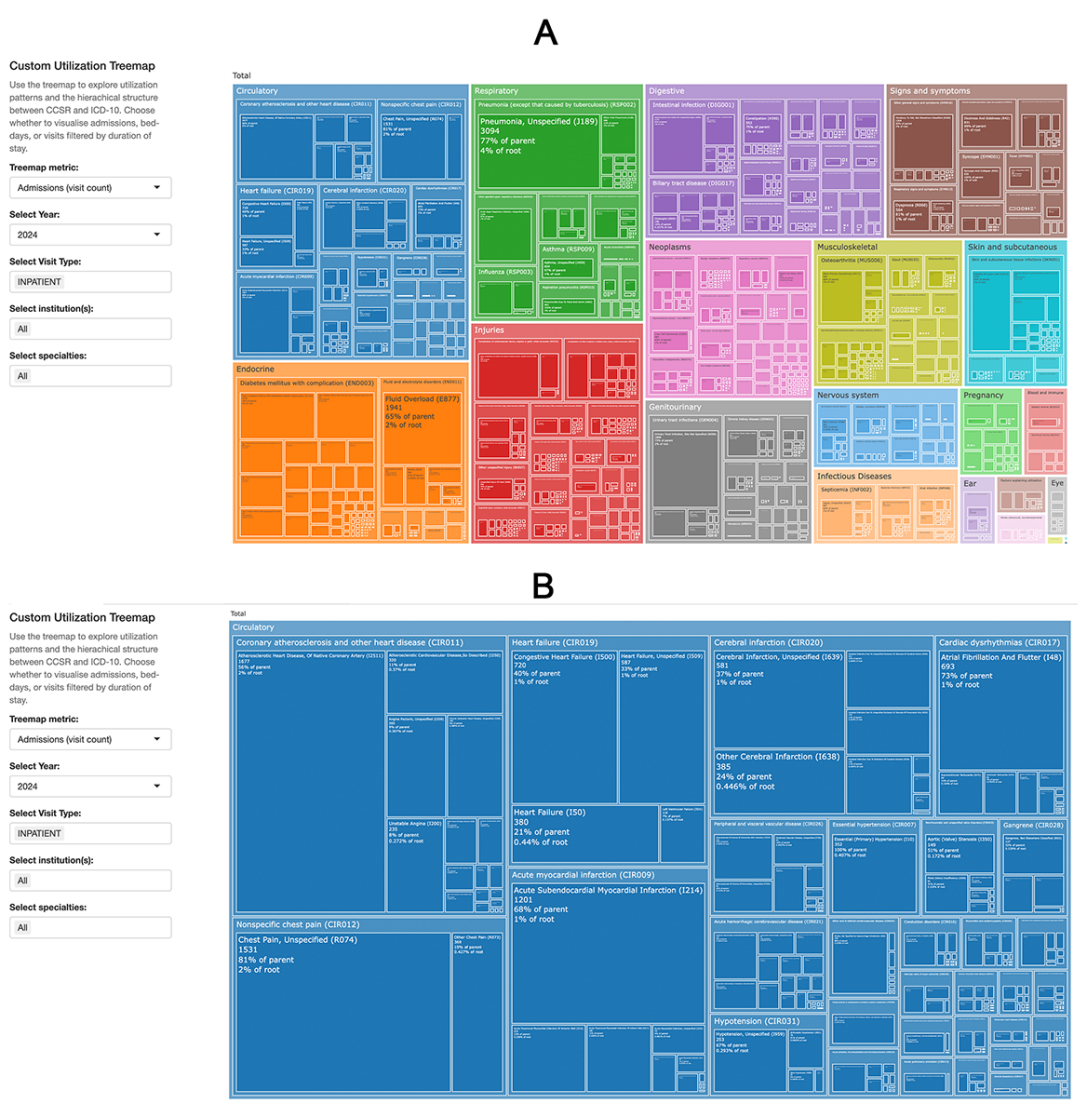
Interactive treemaps depicting inpatient utilization patterns by admission counts in 2024. (A) Overview of all inpatient admissions, visualized by Clinical Classifications Software Refined (CCSR) categories. (B) Expanded view of the circulatory system category, showing corresponding *ICD-10-AM* (*International Classification of Disease, 10th Revision, Australian Modification*) codes nested within each CCSR classification.

CCSR categories and their corresponding *ICD-10-AM* codes were visualized using interactive nested treemaps, enabling users to explore the hierarchical structure of conditions and examine the data at varying levels of detail. These treemaps illustrated the parent-child relationships between CCSR categories and *ICD-10-AM* codes, allowing users to identify high-volume diagnoses and discern patterns across diagnostic groupings. Inpatient utilization was quantified by both admission counts (Figure 1) and total LOS (Figure S3 in Multimedia Appendix 1). Users could apply filters to refine the data view by the LOS categories (in weeks), health care institution, and clinical specialty within our health care cluster. This functionality supported flexible, user-driven analysis of hospitalization patterns across clinical and operational dimensions.

Regarding inpatient utilization by total LOS (Figure S3 in Multimedia Appendix 1), patients in the SDR accounted for 687,062 inpatient bed days in 2024. Circulatory-related CCSR conditions contributed 124,417 (17.7%) bed days, while injuries-related conditions accounted for 86,541 (12.6%) bed days. Among circulatory conditions, the top contributors were CIR020—cerebral infarction, CIR019—heart failure, CIR011—coronary atherosclerosis and other heart diseases, and CIR009—acute myocardial infarction. For injuries-related conditions, the leading contributors were INJ006—fracture of the neck of femur, INJ037—complications of other surgical or medical care, and INJ033—complications related to cardiovascular devices, implants, or grafts.

Inpatient utilization filtered by admission count and LOS provided further insights into conditions associated with prolonged hospitalizations. Figure S4 in Multimedia Appendix 1 presents admissions lasting 4 weeks or more in 2024, totaling 4425 encounters. Of these, circulatory-related conditions accounted for 883 (20.0%) admissions, while injuries-related conditions comprised 658 (14.9%) admissions. Leading circulatory conditions associated with extended stays included CIR020—cerebral infarction, CIR021—acute hemorrhagic cerebrovascular disease, and CIR009—acute myocardial infarction. For injuries-related conditions, top contributors were fracture of the INJ006—neck of femur, INJ037—complications of other surgical or medical care, and INJ005—fractures of the lower limb excluding hip fractures.

### Visualizing Inpatient Utilization, Process Indicators, and Health Status Indicators

**Figure 2.**
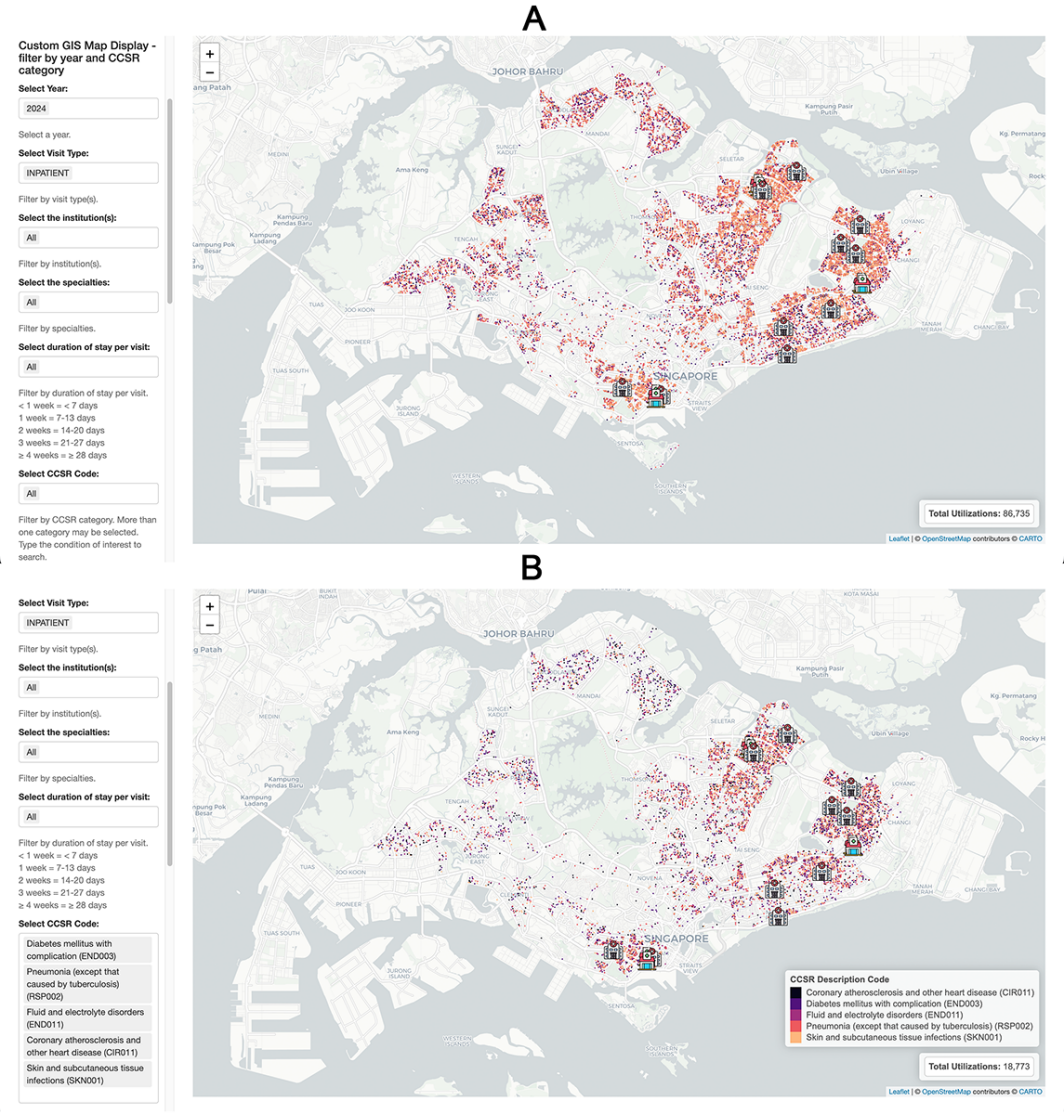
Geospatial bubble maps illustrating inpatient utilization based on the residential addresses of patients in 2024. SingHealth’s acute hospitals, community hospitals, and polyclinics are represented as icons. (A) All Clinical Classification Software Refined (CCSR) categories included. (B) Geospatial map with top 5 CCSR categories for 2024.

The CCSR framework enabled the geospatial visualization of inpatient utilization patterns by residential location (Figure 2). Figure 2A displays utilization in 2024 for all patients in the SDR across all CCSR categories, highlighting that while the density of inpatient utilization was higher in areas within the SingHealth catchment, patients also originated from residential precincts across the island, including those served by other health care clusters. Figure 2B illustrates utilization among patients with the top 5 CCSR categories.

**Figure 3.**
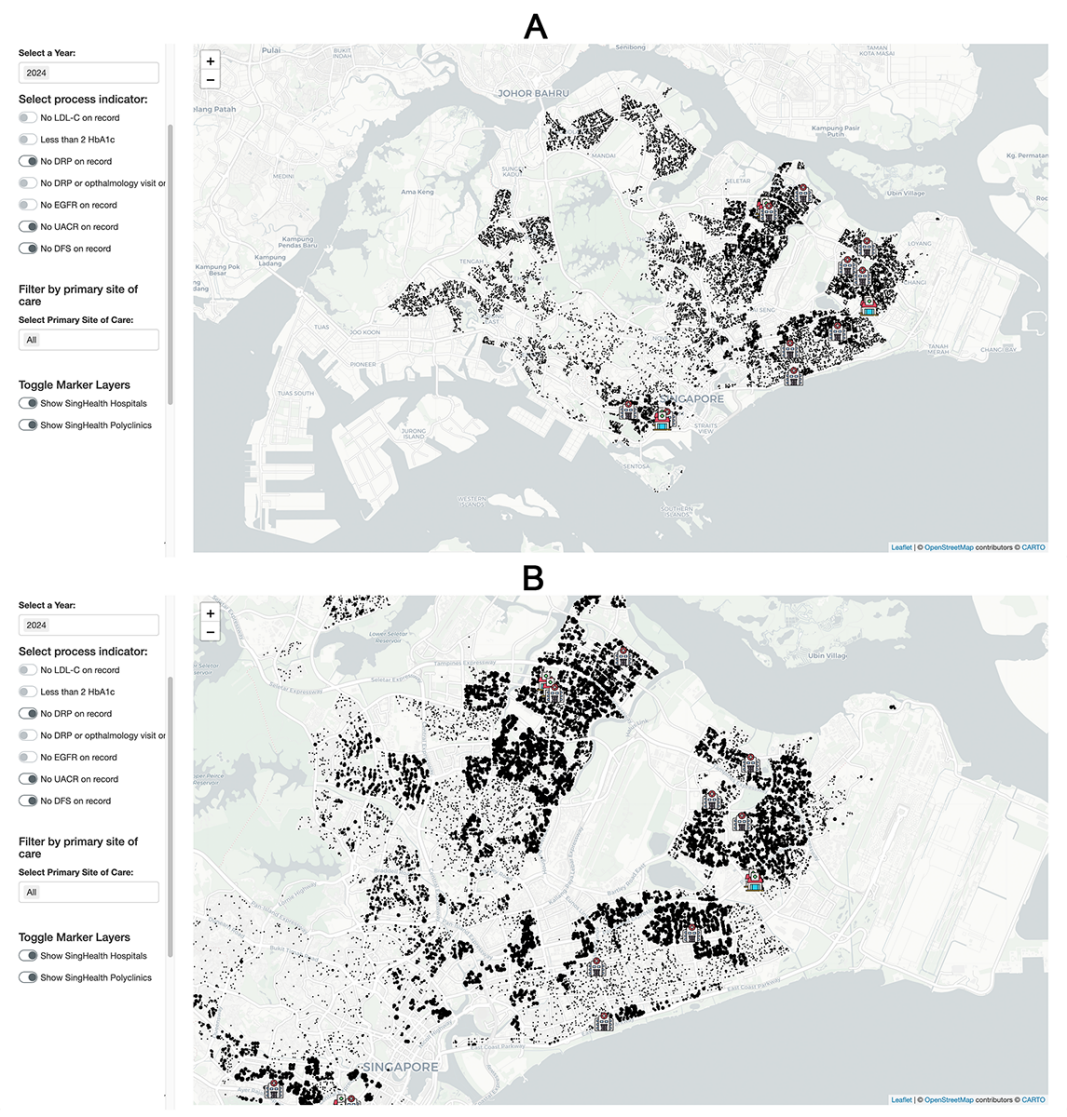
Geospatial bubble maps depicting the residential distribution of patients who did not meet selected diabetes-related process indicators in 2024. Each black bubble represents a geographic cluster of patients missing documentation for all of the following: diabetic retinal photography (DRP), urine albumin-to-creatinine ratio (uACR), and diabetic foot screening (DFS). The size of the bubble reflects the number of affected patients at that location. (A) Full view of Singapore. (B) Zoomed-in view of the southeastern region.

The dashboard enabled the geospatial visualization of patients with unmet diabetes-related process indicators. Figure 3 illustrates this for 2024, highlighting patients without documentation for diabetic retinal photography, urine albumin-to-creatinine ratio, and diabetic foot screening. Each black bubble represents a cluster of patients residing in the same area with all 3 indicators unmet. Panel A displays the nationwide distribution, whereas panel B zooms into the southeastern region.

Prominent clusters were observed in high-density residential zones in the east and northeast, suggesting care delivery gaps and opportunities for targeted community outreach, screening initiatives, and enhanced primary care engagement.

**Figure 4.**
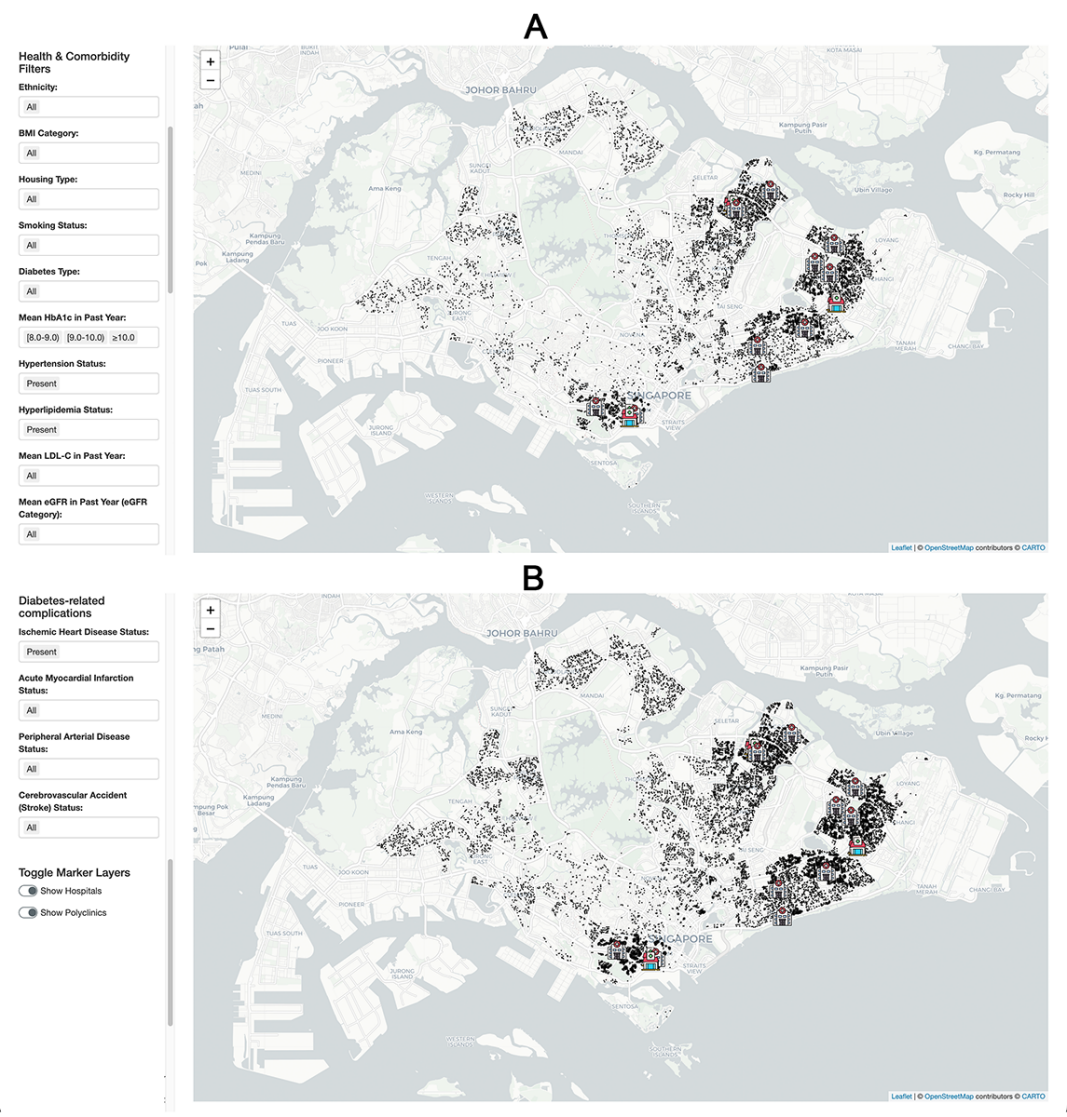
Geospatial bubble maps showing the residential distribution of patients with selected health indicators and diabetes-related complications in 2024. Each black dot represents a cluster of patients residing in the same geographic area. (A) Map selected for patients with HbA_1c_≥8.0%, coexisting with hypertension and hyperlipidemia. (B) Map selected for patients with documented ischemic heart disease.

The dashboard also mapped individuals with poor health status and diabetes-related complications. In Figure 4, panel A displays individuals with suboptimal cardiometabolic control (HbA_1c_ ≥ 8.0%) and coexisting hypertension and hyperlipidemia. Panel B depicts the spatial distribution of patients with prevalent ischemic heart disease, illustrating geographic variation in chronic disease burden.

These interactive maps allow users to zoom to the postal code level, apply filters by health indicators and comorbidities, and overlay health care and community resources—facilitating data-driven, localized planning and intervention design.

## Discussion

### Principal Results

Health care data are inherently complex, often exhibiting hierarchical, relational, and temporal dimensions. While modern health information systems capture large volumes of data, transforming them into actionable insights that support impactful interventions remains a persistent challenge. In this study, we applied the CCSR framework to organize *ICD-10-AM* primary discharge codes into clinically meaningful categories, then leveraged interactive treemaps to facilitate exploration of inpatient utilization patterns, particularly for high-burden conditions while retaining the hierarchical relationships between CCSR categories and *ICD-10-AM* codes. In parallel, we employed GIS mapping to display spatial patterns in inpatient utilization, care process adherence, and health status indicators, enabling interactive, user-driven exploration and regional customization.

### Comparison With Prior Work and Contribution to the Field

While health care dashboards are widely used [10], the literature on dashboards specifically designed for PHM remains limited. In Queensland, Australia, a proof-of-concept dashboard was developed using synthetic data to demonstrate the potential of routinely collected electronic health records for geospatial, descriptive, and longitudinal analytics on obesity surveillance [12]. In the United States, the Veterans Affairs system implemented a national heart failure dashboard focused on administrative tracking and quality monitoring across its patient population [13]. Although both examples highlight the promise of dashboards for informing public health action, they remain limited in scope—either focusing on a single condition or lacking integration across multiple domains of care.

Building on this, our dashboard represents a novel application that unifies inpatient utilization data, health status indicators, and care processes into a single platform. It supports multidimensional, area-level analyses through the combined use of the CCSR framework and geospatial mapping. To the best of our knowledge, this is among the first applications of CCSR categories within an interactive GIS dashboard to enable regional assessment of health care utilization and to identify potential priorities for population health planning at both clinical and administrative levels.

Beyond the technical implementation, our experience highlighted several practical considerations relevant to the development of population health dashboards. First, iterative development through sustained stakeholder engagement was highly valuable. Close collaboration among clinicians, technical developers, population health planners, and community partners was important to identify the core analytic features and ensure that these were technically feasible, operationally relevant, and aligned with real-world population health workflows. Importantly, dashboard development remained an ongoing and adaptive process, with continued refinement of analytic features and outputs through iterative engagement with stakeholders beyond the period described in this paper.

Second, dashboard outputs needed to align closely with clinical and operational processes to ensure that the information presented was both clinically interpretable and practically actionable. In this regard, engagement with domain experts and operational stakeholders was essential for contextualizing findings and guiding appropriate interpretation of the data. Finally, user experience and interface design were critical in balancing analytical complexity with usability for nontechnical users, particularly for stakeholders involved in operational planning and community-based care delivery.

### Insights Into Inpatient Utilization

The application of the CCSR framework proved valuable in addressing the granularity of *ICD-10-AM* diagnostic codes by grouping them into clinically meaningful categories. Interactive nested treemaps enabled users to explore the hierarchical relationships between CCSR categories and individual *ICD-10-AM* codes. Categorizing inpatient utilization using 3 complementary metrics—admission counts, total LOS, and admission counts stratified by LOS—allowed health care utilization to be examined through different analytical lenses.

Admission counts provide a broad estimate of disease burden based on the frequency of visits, although they may include repeat visits by the same individual. In contrast, total LOS provides insights into the intensity of resource use, reflecting the cumulative demand placed on finite bed capacity. Stratifying admission counts by LOS further enables the identification of conditions associated with prolonged hospitalizations. Together, these complementary perspectives support a multidimensional understanding of health care utilization, with relevance for population health planning, service delivery optimization, and operational decision-making.

From the perspective of admission frequency, our analysis identified several high-burden conditions, including diabetes-related renal complications, foot ulcers, pneumonia, fluid overload, electrolyte disorders, and skin and subcutaneous tissue infections. In contrast, when examining inpatient utilization based on total bed days and prolonged admissions (≥4 wk), conditions related to circulatory disease and injuries emerged as major contributors. These findings suggest that conditions driving high admission volumes may differ from those associated with the greatest resource intensity, underscoring the importance of considering different utilization metrics when informing health care planning and prioritization.

While this discussion focuses on the most prevalent CCSR categories, less common conditions may also contribute meaningfully to health care resource use and present opportunities for intervention. Continued engagement with stakeholders remains important for identifying underrecognized patterns of utilization and informing future population health planning priorities.

### Geospatial Mapping for Community Targeting and Strategic Planning

GIS mapping enabled the visualization of inpatient utilization patterns, gaps in adherence to recommended process indicators, and areas with poor health status based on the residential locations of patients. These spatial insights supported community-based situational analysis and population health planning. For instance, among the top 5 CCSR conditions in 2024, distinct geographic variation in distribution was observed, suggesting that different regions may have different population health needs and service demands. For example, areas with high rates of diabetes-related complications may benefit from enhanced postdischarge support and community nursing services, while regions with a high burden of pneumonia-related admissions may warrant strengthened partnerships with local private GPs to facilitate follow-up care and delivery of preventive services, including influenza and pneumococcal vaccinations, aligned with national vaccination guidelines [14].

GIS mapping of process indicators also proved valuable for population health operations. By overlaying community-based assets such as partner GP clinics and AACs onto residential areas where patients had not received annual diabetic retinal photography and diabetic foot screening, health care planners were able to identify priority locations for the strategic deployment of screening services. Importantly, prioritization was influenced not only by the concentration of unmet needs but also by the availability and readiness of community assets to support screening and follow-up activities. This alignment of need and capacity was important to ensure that proposed initiatives were feasible within the existing community infrastructure.

Similarly, GIS mapping of health status indicators enabled planners to identify geographic areas with suboptimal diabetes control, abnormal biomarker profiles, or a high burden of diabetes-related complications. These insights informed engagement between community teams and stakeholders, including community nurses, partner GPs, AACs, and grassroots leaders, to support planning discussions. Furthermore, these data have informed infrastructure planning. For example, as SingHealth prepares to establish a new acute hospital in the eastern region of Singapore, spatial analyses of inpatient utilization and population health metrics are being used to support service planning and resource allocation considerations for the new facility.

These experiences highlight the dashboard’s utility for situational analysis and operations planning. Future development could extend its use toward longitudinal monitoring of health status, process adherence, and utilization patterns across geographic regions over time. Such analyses may help health care planners evaluate the effects of targeted interventions on specific health outcomes and support data-informed decision-making.

### Limitations

This study has several limitations. First, our analysis focused on primary *ICD-10-AM* diagnosis codes and did not incorporate secondary diagnoses. While secondary diagnosis codes may provide additional clinical context—particularly regarding comorbidities and contributing conditions—their inclusion would substantially increase analytical complexity due to their volume and variability in coding practices. In addition, the completeness and consistency of secondary diagnosis coding may differ across encounters and institutions, potentially introducing bias. Further methodological work is needed to determine how secondary diagnosis data can be systematically incorporated to generate reliable and actionable insights for population health analysis.

The *ICD-10-AM* to CCSR mapping process also has inherent limitations. Although we used a semiautomated approach informed by the *ICD-10* hierarchical structure and the published CCSR framework, a proportion of codes mapped to multiple potential CCSR categories and required manual clinical review. While these mappings were independently reviewed by clinicians and resolved through consensus using official CCSR guidance documentation, we did not formally assess interrater reliability, conduct external validation, or perform quantitative accuracy testing of the final mappings. As such, some degree of misclassification may remain. Nevertheless, we believe that the mapping approach provided a pragmatic and clinically meaningful framework for exploratory population health analysis in a non-US *ICD-10-AM* setting. Future work should explore more formal validation approaches and reproducibility assessments to strengthen generalizability and methodological robustness.

A key limitation of this study is data latency, as the dashboard currently reflects data that lag behind real time by approximately 1 year. Because health care utilization patterns evolve, patients requiring inpatient care in 1 year may not do so in subsequent years [15]. Ongoing enhancements to our data pipeline may help reduce data latency, and the future incorporation of predictive analytics could further enable more timely and anticipatory interventions [16].

Nevertheless, data latency does not negate the utility of the dashboard. Our analyses of health status and process indicators from 2019 to 2024 demonstrate that geographic areas with persistent gaps in adherence to recommended care processes and consistently poorer health status recur over time. These stable spatial patterns provide a strong empirical basis for prioritizing targeted, place-based interventions and underscore the opportunity for health systems to act decisively on longitudinal insights while continuing to evolve toward more real-time, proactive population health intelligence.

Our study faced practical data-related limitations, as it relied on the SDR—a mature and comprehensively maintained chronic disease data source within our institution. As a result, the dashboard was developed within a single health care cluster in Singapore and focused exclusively on patients with diabetes mellitus. This may limit generalizability to other populations, disease areas, or international contexts. Adapting the dashboard for other settings would require alignment with local coding systems, availability of structured registry or electronic medical record data, and inclusion of contextually relevant indicators. As more quality-assured data repositories become available, the dashboard’s modular architecture and use of open-source tools will support broader adoption through appropriate customization.

Another important limitation is that the dashboard does not currently incorporate data from the private primary care sector, restricting its ability to reflect care delivered outside the public health care system. Under Healthier SG [17]—Singapore’s national population health initiative—participating GPs are required to use electronic clinic management systems that automatically share selected clinical information with the National Electronic Health Record [18]. These data are subsequently made available by the Ministry of Health to health care clusters and community partners to support outreach and care coordination.

However, Healthier SG primary care data are not yet linked to the SDR or integrated into the dashboard. Ongoing efforts aim to enable this connection, which would significantly enhance the dashboard’s ability to support whole-of-system PHM by providing a more complete view of care across settings. Achieving this will require sustained collaboration and policy support to bridge data silos, enable secure cross-sector information sharing, and align data governance and use frameworks. These system-level enablers are essential for unlocking the full potential of digital tools for coordinated, data-driven population health planning.

Additionally, in accordance with current ethical and data governance requirements, the dashboard is built using deidentified patient-level data that cannot be traced back to individuals. Access to the dashboard is restricted to authorized users, and full addresses and postal codes for single-unit housing (ie, nonapartment residences) are masked across all user views. At the same time, these protections introduce practical limitations to the precision of PHM strategies. While deidentified data are highly valuable for situational analysis, regional planning, and identification of broad population-level patterns, the implementation of targeted interventions—especially those requiring follow-up at the individual level—may require carefully governed access to identifiable data under appropriate ethical, legal, and operational frameworks.

We are mindful of the ethical and governance complexities this entails. Issues of privacy, consent, autonomy, and the risk of overly paternalistic approaches must be carefully considered. Striking the right balance between respecting individuals’ rights and enabling timely, data-informed health interventions is an ongoing ethical and conceptual challenge. Addressing this tension will require the development of robust frameworks that extend beyond technical solutions, encompassing ethical deliberation, stakeholder engagement, and policy dialogue.

Finally, while the dashboard was developed through iterative feedback from stakeholders, no structured usability testing, user surveys, or outcome-based assessments were conducted. Future work should incorporate systematic evaluation frameworks to assess how the dashboard influences user behavior, supports care planning, and drives improvements in population health outcomes.

### Conclusion

The dashboard developed in this study provides meaningful insights into the health status, care processes, and health care utilization patterns of patients with diabetes within our health system. By integrating these indicators with geospatial visualization, the platform enables rich clinical and operational analyses to support data-informed PHM. Ongoing collaboration with stakeholders—and the planned incorporation of predictive and prescriptive analytics—will further extend its utility by supporting more proactive, targeted, and equitable interventions.

## Supplementary material

10.2196/80431 Multimedia Appendix 1 Supplementary methodological figures, inpatient utilization visualizations, and annotated R code for the *International Classification of Diseases, 10th Revision, Australian Modification* to Clinical Classifications Software Refined mapping algorithm.

## Abbreviations

AAC: active aging center
CCSR: Clinical Classification Software Refined
GIS: geographic information system
GP: general practitioner
*ICD-10*: *International Classification of Disease, 10th Revision*
*ICD-10-AM*: *International Classification of Disease, 10th Revision, Australian Modification*
*ICD-10-CM*: *International Classification of Disease, 10th Revision, Clinical Modification*
LOS: length of stay
PHM: population health management
SDR: SingHealth Diabetes Registry
UI: user interface
