# Supplementary material for: Integrating Clinical Classifications Software Refined, Process Indicators, and Geographic Information System Mapping to Inform Population Health Management: Development of an Interactive Dashboard
Source: JMIR Med Inform. 2026 Jul 15;14:e80431. doi: 10.2196/80431 (PMC13372073; doi:10.2196/80431)
Supplement: Multimedia Appendix 1 [file medinform-v14-e80431-s001.docx]

**Multimedia Appendix 1**

**Contents**

**Figure S1**. Schematic of the ICD-10-AM to CCSR matching algorithm.

**Figure S2.** Flow chart for the mapping of ICD-10-AM codes to ICD-10-CM codes.

**Figure S3.** Interactive treemaps depicting inpatient utilization patterns quantified by length of stay in 2024. (A) Overall inpatient utilization. (B) Inpatient utilization for circulatory related CCSR conditions. (C) Inpatient utilization for injuries related CCSR conditions.

**Figure S4.** Interactive treemaps depicting inpatient utilization patterns quantified by admission count and filtered for visits ≥ 4 weeks in 2024. (A) Overall inpatient utilization. (B) Inpatient utilization for circulatory related CCSR conditions. (C) Inpatient utilization for injuries related CCSR conditions.

**Box 1.** R code for iteratively matching ICD-10-AM to CCSR.


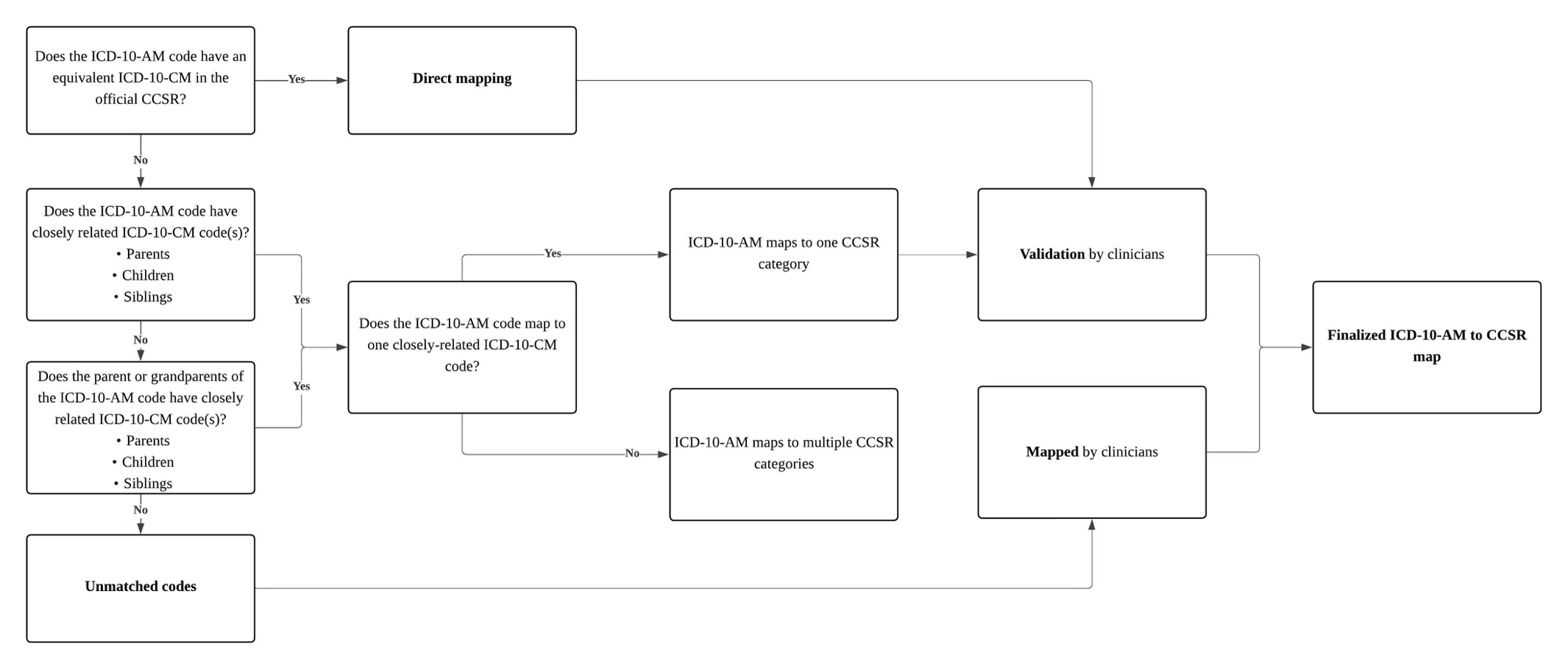


**Figure S1**. Schematic of the ICD-10-AM to CCSR matching algorithm.


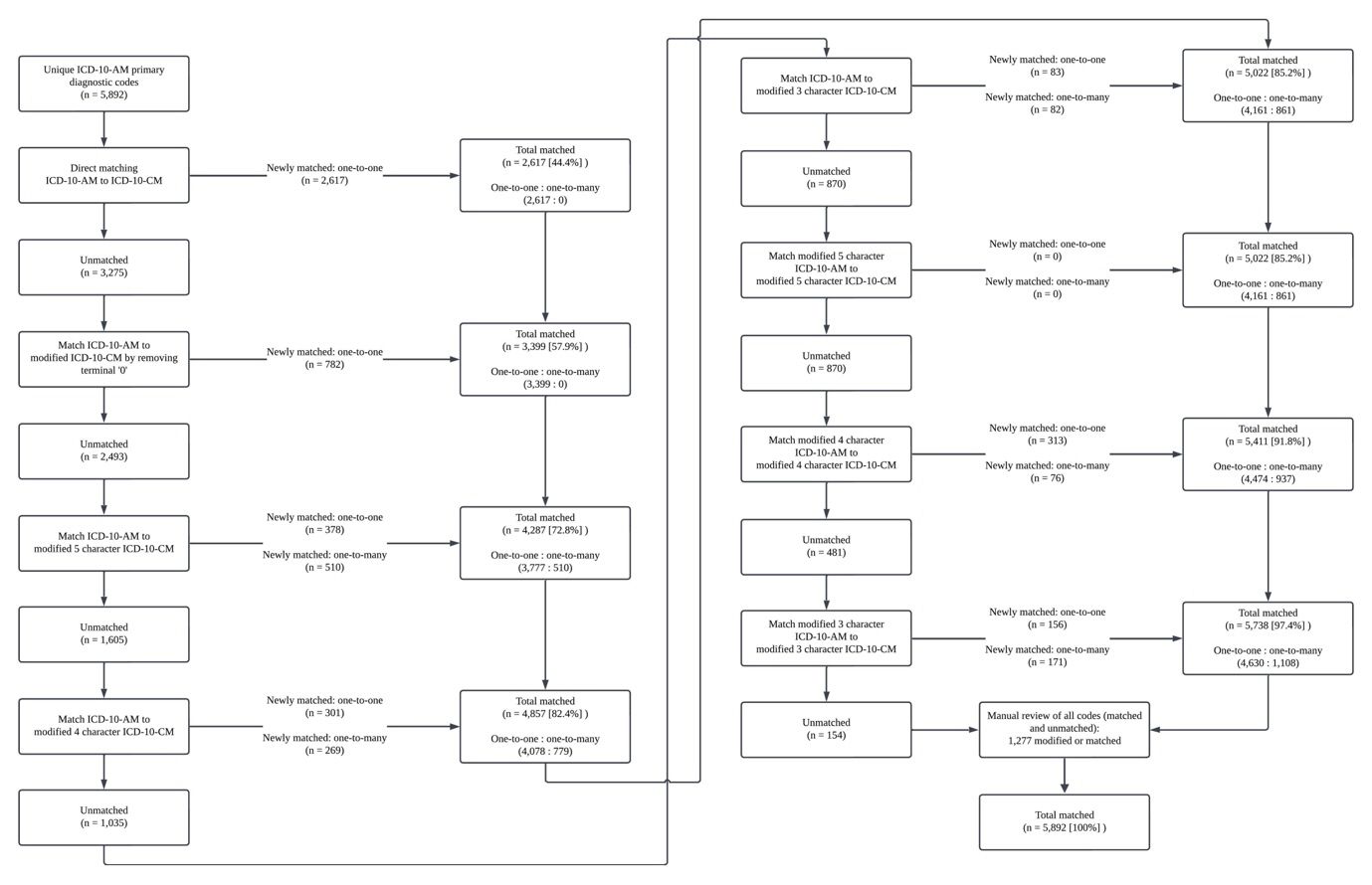


**Figure S2.** Flow chart for the mapping of ICD-10-AM codes to ICD-10-CM codes.

**
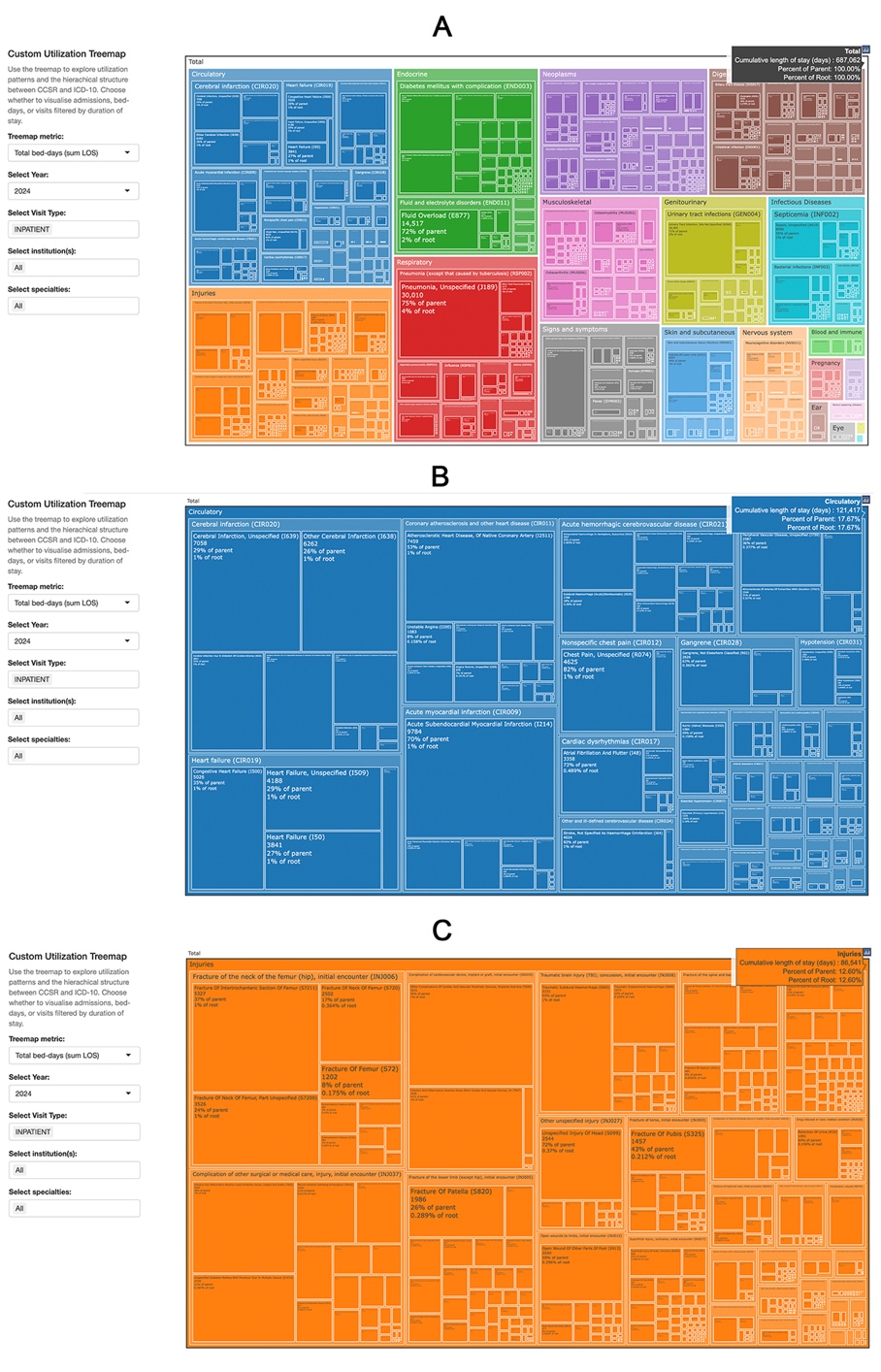
**

**Figure S3.** Interactive treemaps depicting inpatient utilization patterns quantified by length of stay in 2024. (A) Overall inpatient utilization. (B) Inpatient utilization for circulatory related CCSR conditions. (C) Inpatient utilization for injuries related CCSR conditions.


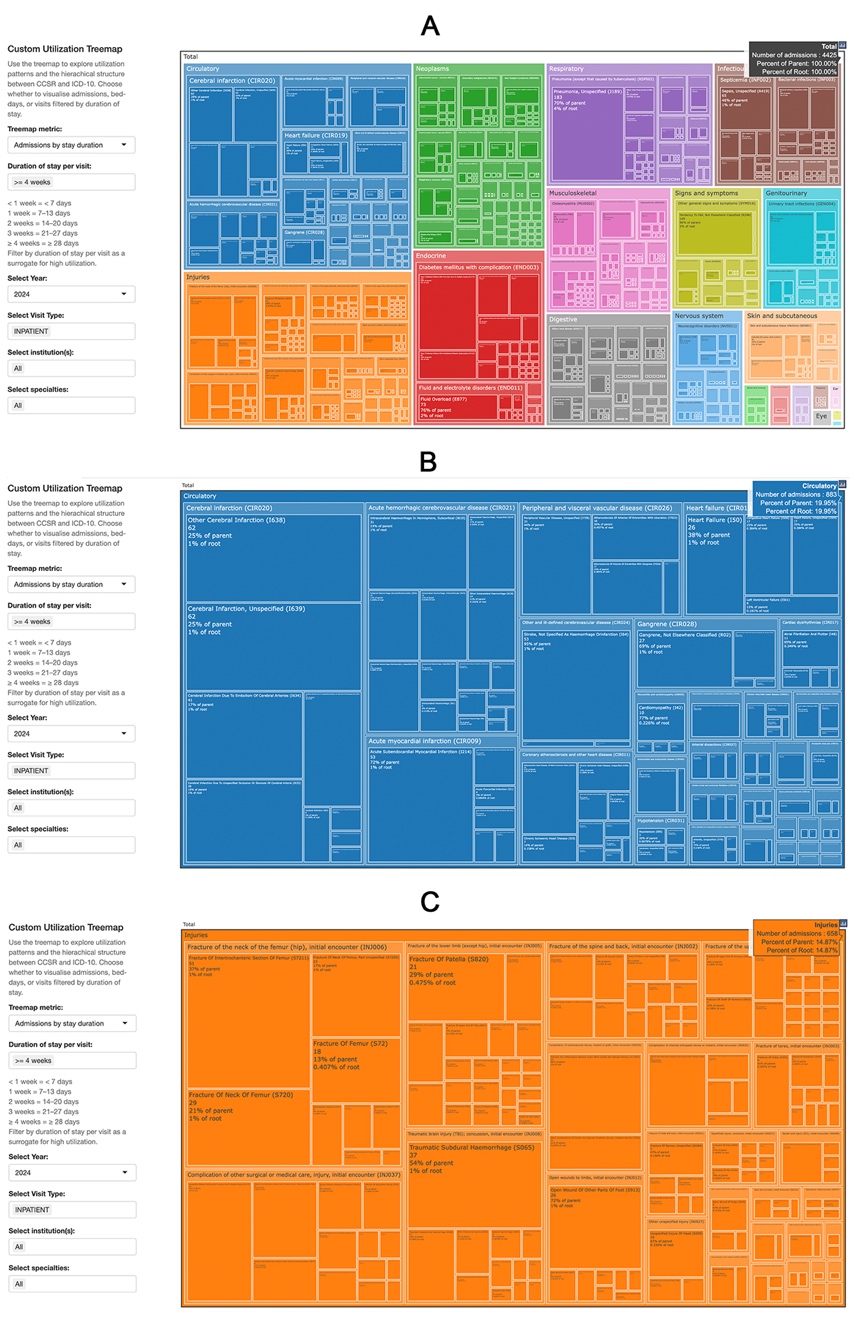


**Figure S4.** Interactive treemaps depicting inpatient utilization patterns quantified by admission count and filtered for visits ≥ 4 weeks in 2024. (A) Overall inpatient utilization. (B) Inpatient utilization for circulatory related CCSR conditions. (C) Inpatient utilization for injuries related CCSR conditions.

| **Box 1.** R code for iteratively matching ICD-10-AM to CCSR. |
| --- |
| # --------------------------------------------------------------------------------------  # Purpose:  # This R script automates the mapping of ICD-10-AM codes to CCSR categories (v2025.1).  # Assumption: ICD-10-AM codes differ in structure from ICD-10-CM; hence the iterative 5-, 4-, 3-character approximation strategy.  # It applies an iterative approximation strategy using 5-, 4-, and 3-character matches.  # One-to-one and one-to-many mappings are handled and reviewed for completeness.  # The final output includes ICD-10-AM codes with corresponding CCSR category and description.  # --------------------------------------------------------------------------------------  library(dplyr)  library(tidyr)  library(readxl)  library(stringr)  ### Step 1. Import data files  # Import ICD-10-AM codes  unique_ICD_codes <- read.csv(file = 'unique_ICD_10_AM_codes.csv')  unique_ICD_codes <- unique_ICD_codes %>% distinct(DIAGNOSIS_CODE, .keep_all = TRUE) # Retain only unique ICD-10 codes  # Import CCSR csv file (from "DXCCSR_v2025-1.csv")  CCSR <- read.csv('DXCCSR_v2025-1.csv')  # Import CCSR category description (from "DXCCSR-Reference-File-v2025-1.xlsx)  CCSR_categories_desc <- read_xlsx(path = 'DXCCSR-Reference-File-v2025-1.xlsx',  sheet = "CCSR_Categories")  CCSR_categories_desc <- CCSR_categories_desc %>%  rename(Default_CCSR_Category = names(CCSR_categories_desc) [1], Default_CCSR_Category_Desc = ...2)  ### Step 2. Modify the CCSR database  ### Step 2.1 Rename columns in original CCSR  # Rename columns for main CCSR  CCSR$X.ICD.10.CM.CODE. <- gsub("'", "", CCSR$X.ICD.10.CM.CODE.)  CCSR <- CCSR %>% rename(ICD_10 = X.ICD.10.CM.CODE.)  CCSR <- CCSR %>% rename(Default_CCSR_Category = X.Default.CCSR.CATEGORY.IP.)  CCSR <- CCSR %>% rename(Default_CCSR_Category_Desc = Default.CCSR.CATEGORY.DESCRIPTION.IP)  # Step 2.2 Modify the ICD codes by removing the last '0' if present  # Duplicate CCSR and modify  CCSR_mod <- CCSR  # Remove trailing '0' from ICD codes to improve matching with ICD-10-AM codes  CCSR_mod$ICD_10 <- sub("0$", "", CCSR_mod$ICD_10)  sum(duplicated(CCSR_mod$ICD_10))  CCSR_mod$Is_Duplicated <- duplicated(CCSR_mod$ICD_10) \| duplicated(CCSR_mod$ICD_10, fromLast = TRUE)  CCSR_mod$ICD_10_concat <- paste(CCSR_mod$ICD_10, CCSR_mod$Default_CCSR_Category, sep = "_")  sum(duplicated(CCSR_mod$ICD_10_concat))  # Remove duplicates from CCSR_mod concatenated values  CCSR_mod_unique <- CCSR_mod %>%  distinct(ICD_10_concat, .keep_all = TRUE)  sum(duplicated(CCSR_mod_unique$ICD_10_concat))  CCSR_mod_unique <- CCSR_mod_unique %>% select(c(ICD_10, Default_CCSR_Category, Default_CCSR_Category_Desc))  # Step 2.3 Modify the ICD codes by reducing it to only 4 characters  # Duplicate CCSR and modify  CCSR_mod2 <- CCSR  # Extract the first 4 characters of the ICD code for approximate (parent-level) matching  CCSR_mod2$ICD_10_4char <- substr(CCSR_mod2$ICD_10, 1, 4)  CCSR_mod2$ICD_10_4char_CCSR <- paste(CCSR_mod2$ICD_10_4char, CCSR_mod2$Default_CCSR_Category, sep = "_")  # Convert CCSR_mod2 into a data frame format  CCSR_mod2 <- CCSR_mod2$ICD_10_4char_CCSR  CCSR_mod2 <- as.data.frame(CCSR_mod2)  # Remove duplicates  CCSR_mod2_unique <- CCSR_mod2 %>% distinct(CCSR_mod2)  sum(duplicated(CCSR_mod2_unique$CCSR_mod2))  # Split strings based on "_" delimiter  CCSR_mod2_split <- strsplit(CCSR_mod2_unique$CCSR_mod2, "_")  # Convert the split results back to a data frame (if needed)  CCSR_mod2_split <- data.frame(matrix(unlist(CCSR_mod2_split), ncol = 2, byrow = TRUE))  CCSR_mod2_split <- CCSR_mod2_split %>% rename(ICD_10 = X1, Default_CCSR_Category = X2)  # Examine CCSR_mod2  sum(duplicated(CCSR_mod2_split$ICD_10))  CCSR_mod2_split$Is_Duplicated <- duplicated(CCSR_mod2_split$ICD_10) \| duplicated(CCSR_mod2_split$ICD_10, fromLast = TRUE)  CCSR_mod2_non_dupli_ICD <- CCSR_mod2_split %>% filter(Is_Duplicated == FALSE)  sum(duplicated(CCSR_mod2_non_dupli_ICD$ICD_10))  # Create a data frame containing CCSR default code and CCSR description  CCSR_default_cat <- CCSR %>% select(c(Default_CCSR_Category, Default_CCSR_Category_Desc))  CCSR_default_cat$Default_cat <- paste(CCSR_default_cat$Default_CCSR_Category, CCSR_default_cat$Default_CCSR_Category_Desc, sep = "_")  CCSR_default_cat <- CCSR_default_cat %>% select(c(Default_cat))  CCSR_default_cat <- CCSR_default_cat %>% distinct(Default_cat)  CCSR_default_cat <- strsplit(CCSR_default_cat$Default_cat, "_")  CCSR_default_cat <- data.frame(matrix(unlist(CCSR_default_cat), ncol = 2, byrow = TRUE))  CCSR_default_cat <- rename(CCSR_default_cat, Default_CCSR_Category = X1, Default_CCSR_Category_Desc = X2)  # Match back the description codes  CCSR_mod2_non_dupli_ICD <- CCSR_mod2_non_dupli_ICD %>% left_join(y = CCSR_default_cat, by = "Default_CCSR_Category")  # Step 2.3 Modify the ICD codes from CCSR_mod2  # Here we use the duplicates from CCSR_mod2 (CCSR_mod2_dupli_ICD) to map single 4-char ICD maps to multiple CCSR code  # Select duplicated codes  CCSR_mod2_dupli_ICD <- CCSR_mod2_split %>% filter(Is_Duplicated == TRUE)  CCSR_mod2_dupli_ICD <- CCSR_mod2_dupli_ICD %>% select(-Is_Duplicated)  # Match back the description codes  CCSR_mod2_dupli_ICD <- CCSR_mod2_dupli_ICD %>% left_join(y = CCSR_default_cat, by = "Default_CCSR_Category")  # Convert CCSR_mod2_duplic_ICD to wide, and thereby resolve the duplicate ICD  # Add a row number for each unique ICD_10 and Default_CCSR_Category combination  CCSR_mod2_dupli_ICD <- CCSR_mod2_dupli_ICD %>%  group_by(ICD_10) %>%  mutate(row_id = row_number()) %>%  ungroup()  # Pivot the data wider, creating new columns for each row_id  CCSR_mod3 <- CCSR_mod2_dupli_ICD %>%  pivot_wider(  id_cols = ICD_10,  names_from = row_id,  values_from = c(Default_CCSR_Category, Default_CCSR_Category_Desc),  names_sep = "_"  )  CCSR_mod3$Default_CCSR_Category <- paste(ifelse(is.na(CCSR_mod3$Default_CCSR_Category_1), "", CCSR_mod3$Default_CCSR_Category_1),  ifelse(is.na(CCSR_mod3$Default_CCSR_Category_2), "", CCSR_mod3$Default_CCSR_Category_2),  ifelse(is.na(CCSR_mod3$Default_CCSR_Category_3), "", CCSR_mod3$Default_CCSR_Category_3),  ifelse(is.na(CCSR_mod3$Default_CCSR_Category_4), "", CCSR_mod3$Default_CCSR_Category_4),  ifelse(is.na(CCSR_mod3$Default_CCSR_Category_5), "", CCSR_mod3$Default_CCSR_Category_5),  ifelse(is.na(CCSR_mod3$Default_CCSR_Category_6), "", CCSR_mod3$Default_CCSR_Category_6),  ifelse(is.na(CCSR_mod3$Default_CCSR_Category_7), "", CCSR_mod3$Default_CCSR_Category_7),  ifelse(is.na(CCSR_mod3$Default_CCSR_Category_8), "", CCSR_mod3$Default_CCSR_Category_8),  ifelse(is.na(CCSR_mod3$Default_CCSR_Category_9), "", CCSR_mod3$Default_CCSR_Category_9)  , sep = " ")  CCSR_mod3$Default_CCSR_Category_Desc <- paste(ifelse(is.na(CCSR_mod3$Default_CCSR_Category_Desc_1), "", CCSR_mod3$Default_CCSR_Category_Desc_1),  ifelse(is.na(CCSR_mod3$Default_CCSR_Category_Desc_2), "", CCSR_mod3$Default_CCSR_Category_Desc_2),  ifelse(is.na(CCSR_mod3$Default_CCSR_Category_Desc_3), "", CCSR_mod3$Default_CCSR_Category_Desc_3),  ifelse(is.na(CCSR_mod3$Default_CCSR_Category_Desc_4), "", CCSR_mod3$Default_CCSR_Category_Desc_4),  ifelse(is.na(CCSR_mod3$Default_CCSR_Category_Desc_5), "", CCSR_mod3$Default_CCSR_Category_Desc_5),  ifelse(is.na(CCSR_mod3$Default_CCSR_Category_Desc_6), "", CCSR_mod3$Default_CCSR_Category_Desc_6),  ifelse(is.na(CCSR_mod3$Default_CCSR_Category_Desc_7), "", CCSR_mod3$Default_CCSR_Category_Desc_7),  ifelse(is.na(CCSR_mod3$Default_CCSR_Category_Desc_8), "", CCSR_mod3$Default_CCSR_Category_Desc_8),  ifelse(is.na(CCSR_mod3$Default_CCSR_Category_Desc_9), "", CCSR_mod3$Default_CCSR_Category_Desc_9)  , sep = " \| ")  CCSR_mod3_final <- CCSR_mod3 %>% select(c(ICD_10, Default_CCSR_Category, Default_CCSR_Category_Desc))  # Step 2.4 Modify the ICD codes by reducing it to only 5 characters  # Duplicate CCSR and modify  CCSR_mod4 <- CCSR  # Extract the first 5 characters  CCSR_mod4$ICD_10_5char <- substr(CCSR_mod4$ICD_10, 1, 5)  CCSR_mod4$ICD_10_5char_CCSR <- paste(CCSR_mod4$ICD_10_5char, CCSR_mod4$Default_CCSR_Category, sep = "_")  # Convert CCSR_mod2 into a data frame format  CCSR_mod4 <- CCSR_mod4$ICD_10_5char_CCSR  CCSR_mod4 <- as.data.frame(CCSR_mod4)  # Remove duplicates  CCSR_mod4_unique <- CCSR_mod4 %>% distinct(CCSR_mod4)  sum(duplicated(CCSR_mod4_unique$CCSR_mod4))  # Split strings based on "_" delimiter  CCSR_mod4_split <- strsplit(CCSR_mod4_unique$CCSR_mod4, "_")  # Convert the split results back to a data frame (if needed)  CCSR_mod4_split <- data.frame(matrix(unlist(CCSR_mod4_split), ncol = 2, byrow = TRUE))  CCSR_mod4_split <- CCSR_mod4_split %>% rename(ICD_10 = X1, Default_CCSR_Category = X2)  # Examine CCSR_mod2  sum(duplicated(CCSR_mod4_split$ICD_10))  CCSR_mod4_split$Is_Duplicated <- duplicated(CCSR_mod4_split$ICD_10) \| duplicated(CCSR_mod4_split$ICD_10, fromLast = TRUE)  CCSR_mod4_non_dupli_ICD <- CCSR_mod4_split %>% filter(Is_Duplicated == FALSE)  sum(duplicated(CCSR_mod4_non_dupli_ICD$ICD_10))  # Match back the description codes  CCSR_mod4_non_dupli_ICD <- CCSR_mod4_non_dupli_ICD %>% left_join(y = CCSR_default_cat, by = "Default_CCSR_Category")  # Step 2.5 Modify the ICD codes from CCSR_mod4  # Select duplicated codes  CCSR_mod4_dupli_ICD <- CCSR_mod4_split %>% filter(Is_Duplicated == TRUE)  CCSR_mod4_dupli_ICD <- CCSR_mod4_dupli_ICD %>% select(-Is_Duplicated)  # Match back the description codes, # CCSR_default_cat was previously created  CCSR_mod4_dupli_ICD <- CCSR_mod4_dupli_ICD %>% left_join(y = CCSR_default_cat, by = "Default_CCSR_Category")  # Convert CCSR_mod2_duplic_ICD to wide, and thereby resolve the duplicate ICD  # Add a row number for each unique ICD_10 and Default_CCSR_Category combination  CCSR_mod4_dupli_ICD <- CCSR_mod4_dupli_ICD %>%  group_by(ICD_10) %>%  mutate(row_id = row_number()) %>%  ungroup()  # Pivot the data wider, creating new columns for each row_id  CCSR_mod5 <- CCSR_mod4_dupli_ICD %>%  pivot_wider(  id_cols = ICD_10,  names_from = row_id,  values_from = c(Default_CCSR_Category, Default_CCSR_Category_Desc),  names_sep = "_"  )  CCSR_mod5$Default_CCSR_Category <- paste(ifelse(is.na(CCSR_mod5$Default_CCSR_Category_1), "", CCSR_mod5$Default_CCSR_Category_1),  ifelse(is.na(CCSR_mod5$Default_CCSR_Category_2), "", CCSR_mod5$Default_CCSR_Category_2),  ifelse(is.na(CCSR_mod5$Default_CCSR_Category_3), "", CCSR_mod5$Default_CCSR_Category_3),  ifelse(is.na(CCSR_mod5$Default_CCSR_Category_4), "", CCSR_mod5$Default_CCSR_Category_4),  ifelse(is.na(CCSR_mod5$Default_CCSR_Category_5), "", CCSR_mod5$Default_CCSR_Category_5),  ifelse(is.na(CCSR_mod5$Default_CCSR_Category_6), "", CCSR_mod5$Default_CCSR_Category_6),  ifelse(is.na(CCSR_mod5$Default_CCSR_Category_7), "", CCSR_mod5$Default_CCSR_Category_7),  ifelse(is.na(CCSR_mod5$Default_CCSR_Category_8), "", CCSR_mod5$Default_CCSR_Category_8),  ifelse(is.na(CCSR_mod5$Default_CCSR_Category_9), "", CCSR_mod5$Default_CCSR_Category_9)  , sep = " ")  CCSR_mod5$Default_CCSR_Category_Desc <- paste(ifelse(is.na(CCSR_mod5$Default_CCSR_Category_Desc_1), "", CCSR_mod5$Default_CCSR_Category_Desc_1),  ifelse(is.na(CCSR_mod5$Default_CCSR_Category_Desc_2), "", CCSR_mod5$Default_CCSR_Category_Desc_2),  ifelse(is.na(CCSR_mod5$Default_CCSR_Category_Desc_3), "", CCSR_mod5$Default_CCSR_Category_Desc_3),  ifelse(is.na(CCSR_mod5$Default_CCSR_Category_Desc_4), "", CCSR_mod5$Default_CCSR_Category_Desc_4),  ifelse(is.na(CCSR_mod5$Default_CCSR_Category_Desc_5), "", CCSR_mod5$Default_CCSR_Category_Desc_5),  ifelse(is.na(CCSR_mod5$Default_CCSR_Category_Desc_6), "", CCSR_mod5$Default_CCSR_Category_Desc_6),  ifelse(is.na(CCSR_mod5$Default_CCSR_Category_Desc_7), "", CCSR_mod5$Default_CCSR_Category_Desc_7),  ifelse(is.na(CCSR_mod5$Default_CCSR_Category_Desc_8), "", CCSR_mod5$Default_CCSR_Category_Desc_8),  ifelse(is.na(CCSR_mod5$Default_CCSR_Category_Desc_9), "", CCSR_mod5$Default_CCSR_Category_Desc_9)  , sep = " \| ")  CCSR_mod5_final <- CCSR_mod5 %>% select(c(ICD_10, Default_CCSR_Category, Default_CCSR_Category_Desc))  # Step 2.6 Modify the ICD codes by reducing it to only 3 characters  # Duplicate CCSR and modify  CCSR_mod6 <- CCSR  # Extract the first 3 characters  CCSR_mod6$ICD_10_3char <- substr(CCSR_mod6$ICD_10, 1, 3)  CCSR_mod6$ICD_10_3char_CCSR <- paste(CCSR_mod6$ICD_10_3char, CCSR_mod6$Default_CCSR_Category, sep = "_")  # Extract and ensure CCSR_mod6 is a data frame  CCSR_mod6 <- CCSR_mod6$ICD_10_3char_CCSR  CCSR_mod6 <- as.data.frame(CCSR_mod6)  # Remove duplicates  CCSR_mod6_unique <- CCSR_mod6 %>% distinct(CCSR_mod6)  sum(duplicated(CCSR_mod6_unique$CCSR_mod6))  # Split strings based on "_" delimiter  CCSR_mod6_split <- strsplit(CCSR_mod6_unique$CCSR_mod6, "_")  # Convert the split results back to a data frame (if needed)  CCSR_mod6_split <- data.frame(matrix(unlist(CCSR_mod6_split), ncol = 2, byrow = TRUE))  CCSR_mod6_split <- CCSR_mod6_split %>% rename(ICD_10 = X1, Default_CCSR_Category = X2)  # Examine CCSR_mod2  sum(duplicated(CCSR_mod6_split$ICD_10))  CCSR_mod6_split$Is_Duplicated <- duplicated(CCSR_mod6_split$ICD_10) \| duplicated(CCSR_mod6_split$ICD_10, fromLast = TRUE)  CCSR_mod6_non_dupli_ICD <- CCSR_mod6_split %>% filter(Is_Duplicated == FALSE)  sum(duplicated(CCSR_mod6_non_dupli_ICD$ICD_10))  # Match back the description codes  CCSR_mod6_non_dupli_ICD <- CCSR_mod6_non_dupli_ICD %>% left_join(y = CCSR_default_cat, by = "Default_CCSR_Category")  # Step 2.7 Modify the ICD codes from CCSR_mod2  # Select duplicated codes  CCSR_mod6_dupli_ICD <- CCSR_mod6_split %>% filter(Is_Duplicated == TRUE)  CCSR_mod6_dupli_ICD <- CCSR_mod6_dupli_ICD %>% select(-Is_Duplicated)  # Match back the description codes, # CCSR_default_cat was previously created  CCSR_mod6_dupli_ICD <- CCSR_mod6_dupli_ICD %>% left_join(y = CCSR_default_cat, by = "Default_CCSR_Category")  # Convert CCSR_mod2_duplic_ICD to wide, and thereby resolve the duplicate ICD  # Add a row number for each unique ICD_10 and Default_CCSR_Category combination  CCSR_mod6_dupli_ICD <- CCSR_mod6_dupli_ICD %>%  group_by(ICD_10) %>%  mutate(row_id = row_number()) %>%  ungroup()  # Pivot the data wider, creating new columns for each row_id  CCSR_mod7 <- CCSR_mod6_dupli_ICD %>%  pivot_wider(  id_cols = ICD_10,  names_from = row_id,  values_from = c(Default_CCSR_Category, Default_CCSR_Category_Desc),  names_sep = "_"  )  CCSR_mod7$Default_CCSR_Category <- paste(ifelse(is.na(CCSR_mod7$Default_CCSR_Category_1), "", CCSR_mod7$Default_CCSR_Category_1),  ifelse(is.na(CCSR_mod7$Default_CCSR_Category_2), "", CCSR_mod7$Default_CCSR_Category_2),  ifelse(is.na(CCSR_mod7$Default_CCSR_Category_3), "", CCSR_mod7$Default_CCSR_Category_3),  ifelse(is.na(CCSR_mod7$Default_CCSR_Category_4), "", CCSR_mod7$Default_CCSR_Category_4),  ifelse(is.na(CCSR_mod7$Default_CCSR_Category_5), "", CCSR_mod7$Default_CCSR_Category_5),  ifelse(is.na(CCSR_mod7$Default_CCSR_Category_6), "", CCSR_mod7$Default_CCSR_Category_6),  ifelse(is.na(CCSR_mod7$Default_CCSR_Category_7), "", CCSR_mod7$Default_CCSR_Category_7),  ifelse(is.na(CCSR_mod7$Default_CCSR_Category_8), "", CCSR_mod7$Default_CCSR_Category_8),  ifelse(is.na(CCSR_mod7$Default_CCSR_Category_9), "", CCSR_mod7$Default_CCSR_Category_9)  , sep = " ")  CCSR_mod7$Default_CCSR_Category_Desc <- paste(ifelse(is.na(CCSR_mod7$Default_CCSR_Category_Desc_1), "", CCSR_mod7$Default_CCSR_Category_Desc_1),  ifelse(is.na(CCSR_mod7$Default_CCSR_Category_Desc_2), "", CCSR_mod7$Default_CCSR_Category_Desc_2),  ifelse(is.na(CCSR_mod7$Default_CCSR_Category_Desc_3), "", CCSR_mod7$Default_CCSR_Category_Desc_3),  ifelse(is.na(CCSR_mod7$Default_CCSR_Category_Desc_4), "", CCSR_mod7$Default_CCSR_Category_Desc_4),  ifelse(is.na(CCSR_mod7$Default_CCSR_Category_Desc_5), "", CCSR_mod7$Default_CCSR_Category_Desc_5),  ifelse(is.na(CCSR_mod7$Default_CCSR_Category_Desc_6), "", CCSR_mod7$Default_CCSR_Category_Desc_6),  ifelse(is.na(CCSR_mod7$Default_CCSR_Category_Desc_7), "", CCSR_mod7$Default_CCSR_Category_Desc_7),  ifelse(is.na(CCSR_mod7$Default_CCSR_Category_Desc_8), "", CCSR_mod7$Default_CCSR_Category_Desc_8),  ifelse(is.na(CCSR_mod7$Default_CCSR_Category_Desc_9), "", CCSR_mod7$Default_CCSR_Category_Desc_9)  , sep = " \| ")  CCSR_mod7_final <- CCSR_mod7 %>% select(c(ICD_10, Default_CCSR_Category, Default_CCSR_Category_Desc))  ### Step 3. Match unique_ICD_codes to CCSR category  # Step 3.1 Modify dataframes  unique_ICD_codes <- unique_ICD_codes %>% rename(ICD_10 = DIAGNOSIS_CODE)  # Merge dataframes with original CCSR  unique_ICD_codes_v1 <- merge(unique_ICD_codes, CCSR[, c("ICD_10", "Default_CCSR_Category", "Default_CCSR_Category_Desc")], by = "ICD_10", all.x = TRUE)  ### Step 3.2 Specify a function for matching  # Helper function to populate missing CCSR category fields  # Arguments:  # - admit_data: data frame of ICD-10-AM codes  # - CCSR_data: reference CCSR mapping data  # - common_id: column name to join by  # - target_column: column in admit_data to populate  # - source_column: column in CCSR_data used as the source  populate_na_category <- function(admit_data, CCSR_data, common_id, target_column, source_column) {  # Merge the two datasets with explicit suffixes  merged_data <- admit_data %>%  left_join(CCSR_data, by = common_id, suffix = c(".admit", ".CCSR")) %>%  mutate(  # Dynamically fill target column if it's NA using the source column  !!target_column := ifelse(  is.na(.[[paste0(target_column, ".admit")]]),  .[[paste0(source_column, ".CCSR")]],  .[[paste0(target_column, ".admit")]]  )  ) %>%  # Drop the source column (with the suffix) if desired  select(-all_of(paste0(source_column, ".CCSR")))    # Remove suffixes from the column names for the final output  # colnames(merged_data) <- gsub("\\.admit$", "", colnames(merged_data))    return(merged_data)  }  ### Step 3.3 Match using CCSR_mod_unique  unique_ICD_codes_v2 <- populate_na_category(  admit_data = unique_ICD_codes_v1,  CCSR_data = CCSR_mod_unique,  common_id = "ICD_10", # Common identifier column  target_column = "Default_CCSR_Category", # Column in unique_ICD_codes to populate  source_column = "Default_CCSR_Category" ) # Column in CCSR_mod to fill from  sum(is.na(unique_ICD_codes_v2$Default_CCSR_Category))  sum(is.na(unique_ICD_codes_v2$Default_CCSR_Category.admit))  sum(is.na(unique_ICD_codes_v1$Default_CCSR_Category))  # Step 3.4 Match using CCSR_mod2_non_dupli_ICD  # Duplicate and clean  unique_ICD_codes_v3 <- unique_ICD_codes_v2 %>% select(-c(Default_CCSR_Category.admit, Default_CCSR_Category_Desc.admit))  unique_ICD_codes_v3 <- unique_ICD_codes_v3 %>% rename(Default_CCSR_Category_Desc = Default_CCSR_Category_Desc.CCSR)  unique_ICD_codes_v3 <- populate_na_category(  admit_data = unique_ICD_codes_v3,  CCSR_data = CCSR_mod2_non_dupli_ICD,  common_id = "ICD_10", # Common identifier column  target_column = "Default_CCSR_Category", # Column in unique_ICD_codes to populate  source_column = "Default_CCSR_Category" ) # Column in CCSR_mod to fill from  # Remove duplicates and count remaining unmatched codes  unique_ICD_codes_v3 <- unique_ICD_codes_v3 %>% select(-c(Is_Duplicated))  sum(is.na(unique_ICD_codes_v3$Default_CCSR_Category))  sum(is.na(unique_ICD_codes_v3$Default_CCSR_Category.admit))  # Step 3.5 Match using CCSR_mod3_final  unique_ICD_codes_v4 <- unique_ICD_codes_v3 %>% select(-c(Default_CCSR_Category_Desc.admit, Default_CCSR_Category.admit))  unique_ICD_codes_v4 <- unique_ICD_codes_v4 %>% rename(Default_CCSR_Category_Desc = Default_CCSR_Category_Desc.CCSR)  unique_ICD_codes_v4 <- populate_na_category(  admit_data = unique_ICD_codes_v4,  CCSR_data = CCSR_mod3_final,  common_id = "ICD_10", # Common identifier column  target_column = "Default_CCSR_Category", # Column in unique_ICD_codes to populate  source_column = "Default_CCSR_Category" ) # Column in CCSR_mod to fill from  sum(is.na(unique_ICD_codes_v4$Default_CCSR_Category)) # latest coding  sum(is.na(unique_ICD_codes_v4$Default_CCSR_Category.admit)) # previous coding  # Step 3.6 Match using CCSR_mod4_non_dupli_ICD  # Duplicate and clean  unique_ICD_codes_v5 <- unique_ICD_codes_v4 %>% select(-c(Default_CCSR_Category.admit, Default_CCSR_Category_Desc.admit))  unique_ICD_codes_v5 <- unique_ICD_codes_v5 %>% rename(Default_CCSR_Category_Desc = Default_CCSR_Category_Desc.CCSR)  unique_ICD_codes_v5 <- populate_na_category(  admit_data = unique_ICD_codes_v5,  CCSR_data = CCSR_mod4_non_dupli_ICD,  common_id = "ICD_10", # Common identifier column  target_column = "Default_CCSR_Category", # Column in unique_ICD_codes to populate  source_column = "Default_CCSR_Category" ) # Column in CCSR_mod to fill from  # Remove duplicates and count remaining unmatched codes  unique_ICD_codes_v5 <- unique_ICD_codes_v5 %>% select(-c(Is_Duplicated))  sum(is.na(unique_ICD_codes_v5$Default_CCSR_Category)) # latest coding  sum(is.na(unique_ICD_codes_v5$Default_CCSR_Category.admit)) # previous coding  # Step 3.7 Match using CCSR_mod5_final  unique_ICD_codes_v6 <- unique_ICD_codes_v5 %>% select(-c(Default_CCSR_Category.admit, Default_CCSR_Category_Desc.admit))  unique_ICD_codes_v6 <- unique_ICD_codes_v6 %>% rename(Default_CCSR_Category_Desc = Default_CCSR_Category_Desc.CCSR)  unique_ICD_codes_v6 <- populate_na_category(  admit_data = unique_ICD_codes_v6,  CCSR_data = CCSR_mod5_final,  common_id = "ICD_10", # Common identifier column  target_column = "Default_CCSR_Category", # Column in unique_ICD_codes to populate  source_column = "Default_CCSR_Category" ) # Column in CCSR_mod to fill from  sum(is.na(unique_ICD_codes_v6$Default_CCSR_Category)) # latest coding  sum(is.na(unique_ICD_codes_v6$Default_CCSR_Category.admit)) # Previous coding  # Step 3.8 Match using CCSR_mod6_non_dupli_ICD  # Duplicate and clean  unique_ICD_codes_v7 <- unique_ICD_codes_v6 %>% select(-c(Default_CCSR_Category.admit, Default_CCSR_Category_Desc.admit))  unique_ICD_codes_v7 <- unique_ICD_codes_v7 %>% rename(Default_CCSR_Category_Desc = Default_CCSR_Category_Desc.CCSR)  unique_ICD_codes_v7 <- populate_na_category(  admit_data = unique_ICD_codes_v7,  CCSR_data = CCSR_mod6_non_dupli_ICD,  common_id = "ICD_10", # Common identifier column  target_column = "Default_CCSR_Category", # Column in unique_ICD_codes to populate  source_column = "Default_CCSR_Category" ) # Column in CCSR_mod to fill from  # Remove duplicates and count remaining unmatched codes  unique_ICD_codes_v7 <- unique_ICD_codes_v7 %>% select(-c(Is_Duplicated))  sum(is.na(unique_ICD_codes_v7$Default_CCSR_Category)) # latest coding  sum(is.na(unique_ICD_codes_v7$Default_CCSR_Category.admit)) # previous coding  # Step 3.9 Match using CCSR_mod7_final  unique_ICD_codes_v8 <- unique_ICD_codes_v7 %>% select(-c(Default_CCSR_Category.admit, Default_CCSR_Category_Desc.admit))  unique_ICD_codes_v8 <- unique_ICD_codes_v8 %>% rename(Default_CCSR_Category_Desc = Default_CCSR_Category_Desc.CCSR)  unique_ICD_codes_v8 <- populate_na_category(  admit_data = unique_ICD_codes_v8,  CCSR_data = CCSR_mod7_final,  common_id = "ICD_10", # Common identifier column  target_column = "Default_CCSR_Category", # Column in unique_ICD_codes to populate  source_column = "Default_CCSR_Category" ) # Column in CCSR_mod to fill from  sum(is.na(unique_ICD_codes_v8$Default_CCSR_Category)) # latest coding  sum(is.na(unique_ICD_codes_v8$Default_CCSR_Category.admit)) # Previous coding  ## Summary of modified CCSR ##  # 4 character non-duplicated (one-to-one) with description -> CCSR_mod2_non_dupli_ICD  CCSR_mod2_non_dupli_ICD$ICD_10_4char <- CCSR_mod2_non_dupli_ICD$ICD_10  # 4 character non-duplicated (one-to-many) with description -> CCSR_mod3_final  CCSR_mod3_final$ICD_10_4char <- CCSR_mod3_final$ICD_10  # 5 character non-duplicated (one-to-one) with description -> CCSR_mod4_non_dupli_ICD  CCSR_mod4_non_dupli_ICD$ICD_10_5char <- CCSR_mod4_non_dupli_ICD$ICD_10  # 5 character non-duplicated (one-to-many) with description -> CCSR_mod5_final  CCSR_mod5_final$ICD_10_5char <- CCSR_mod5_final$ICD_10  # 3 character non-duplicated (one-to-one) with description -> CCSR_mod6_non_dupli_ICD  CCSR_mod6_non_dupli_ICD$ICD_10_3char <- CCSR_mod6_non_dupli_ICD$ICD_10  # 3 character non-duplicated (one-to-many) with description -> CCSR_mod7_final  CCSR_mod7_final$ICD_10_3char <- CCSR_mod7_final$ICD_10  # Step 3.10 Modify unique_ICD_code for matching  unique_ICD_codes_v9 <- unique_ICD_codes_v8 %>% select(-c(Default_CCSR_Category.admit, Default_CCSR_Category_Desc.admit))  unique_ICD_codes_v9 <- unique_ICD_codes_v9 %>% rename(Default_CCSR_Category_Desc = Default_CCSR_Category_Desc.CCSR)  unique_ICD_codes_v9$ICD_10_5char <- substr(unique_ICD_codes_v9$ICD_10, 1, 5) # New variable reducing ICD-10 codes to 5 character only  unique_ICD_codes_v9$ICD_10_4char <- substr(unique_ICD_codes_v9$ICD_10, 1, 4) # New variable reducing ICD-10 codes to 4 character only  unique_ICD_codes_v9$ICD_10_3char <- substr(unique_ICD_codes_v9$ICD_10, 1, 3) # New variable reducing ICD-10 codes to 3 character only  # Step 3.10.1  # 5 character non-duplicated (one-to-one) with description -> CCSR_mod4_non_dupli_ICD  unique_ICD_codes_v10 <- populate_na_category(  admit_data = unique_ICD_codes_v9,  CCSR_data = CCSR_mod4_non_dupli_ICD,  common_id = "ICD_10_5char", # Common identifier column  target_column = "Default_CCSR_Category", # Column in unique_ICD_codes to populate  source_column = "Default_CCSR_Category" ) # Column in CCSR_mod to fill from  sum(is.na(unique_ICD_codes_v10$Default_CCSR_Category)) # latest coding  sum(is.na(unique_ICD_codes_v10$Default_CCSR_Category.admit)) # Previous coding  unique_ICD_codes_v10 <- unique_ICD_codes_v10 %>% select(-c(ICD_10.CCSR, Default_CCSR_Category_Desc.CCSR, Default_CCSR_Category.admit, Default_CCSR_Category_Desc.admit, Is_Duplicated))  # Step 3.10.2  # 5 character non-duplicated (one-to-many) with description -> CCSR_mod5_final  unique_ICD_codes_v11 <- populate_na_category(  admit_data = unique_ICD_codes_v10,  CCSR_data = CCSR_mod5_final,  common_id = "ICD_10_5char", # Common identifier column  target_column = "Default_CCSR_Category", # Column in unique_ICD_codes to populate  source_column = "Default_CCSR_Category" ) # Column in CCSR_mod to fill from  sum(is.na(unique_ICD_codes_v11$Default_CCSR_Category)) # latest coding  sum(is.na(unique_ICD_codes_v11$Default_CCSR_Category.admit)) # Previous coding  unique_ICD_codes_v11 <- unique_ICD_codes_v11 %>% select(-c(Default_CCSR_Category.admit, ICD_10, Default_CCSR_Category_Desc)) # retain original ICD-10 code as ICD_10.admit  # Step 3.10.3  # 4 character non-duplicated (one-to-one) with description -> CCSR_mod2_non_dupli_ICD  unique_ICD_codes_v12 <- populate_na_category(  admit_data = unique_ICD_codes_v11,  CCSR_data = CCSR_mod2_non_dupli_ICD,  common_id = "ICD_10_4char", # Common identifier column  target_column = "Default_CCSR_Category", # Column in unique_ICD_codes to populate  source_column = "Default_CCSR_Category" ) # Column in CCSR_mod to fill from  sum(is.na(unique_ICD_codes_v12$Default_CCSR_Category)) # latest coding  sum(is.na(unique_ICD_codes_v12$Default_CCSR_Category.admit)) # Previous coding  unique_ICD_codes_v12 <- unique_ICD_codes_v12 %>% select(-c(Default_CCSR_Category.admit, ICD_10, Is_Duplicated, Default_CCSR_Category_Desc))  # Step 3.10.4  # 4 character non-duplicated (one-to-many) with description -> CCSR_mod3_final  unique_ICD_codes_v13 <- populate_na_category(  admit_data = unique_ICD_codes_v12,  CCSR_data = CCSR_mod3_final,  common_id = "ICD_10_4char", # Common identifier column  target_column = "Default_CCSR_Category", # Column in unique_ICD_codes to populate  source_column = "Default_CCSR_Category" ) # Column in CCSR_mod to fill from  sum(is.na(unique_ICD_codes_v13$Default_CCSR_Category)) # latest coding  sum(is.na(unique_ICD_codes_v13$Default_CCSR_Category.admit)) # Previous coding  unique_ICD_codes_v13 <- unique_ICD_codes_v13 %>% select(-c(Default_CCSR_Category.admit, ICD_10, Default_CCSR_Category_Desc))  # Step 3.10.5  # 3 character non-duplicated (one-to-one) with description -> CCSR_mod6_non_dupli_ICD  unique_ICD_codes_v14 <- populate_na_category(  admit_data = unique_ICD_codes_v13,  CCSR_data = CCSR_mod6_non_dupli_ICD,  common_id = "ICD_10_3char", # Common identifier column  target_column = "Default_CCSR_Category", # Column in unique_ICD_codes to populate  source_column = "Default_CCSR_Category" ) # Column in CCSR_mod to fill from  sum(is.na(unique_ICD_codes_v14$Default_CCSR_Category)) # latest coding  sum(is.na(unique_ICD_codes_v14$Default_CCSR_Category.admit)) # Previous coding  unique_ICD_codes_v14 <- unique_ICD_codes_v14 %>% select(-c(Default_CCSR_Category.admit, ICD_10, Is_Duplicated, Default_CCSR_Category_Desc))  # Step 3.8.5  # 3 character non-duplicated (one-to-many) with description -> CCSR_mod7_final  unique_ICD_codes_v15 <- populate_na_category(  admit_data = unique_ICD_codes_v14,  CCSR_data = CCSR_mod7_final,  common_id = "ICD_10_3char", # Common identifier column  target_column = "Default_CCSR_Category", # Column in unique_ICD_codes to populate  source_column = "Default_CCSR_Category" ) # Column in CCSR_mod to fill from  sum(is.na(unique_ICD_codes_v15$Default_CCSR_Category)) # latest coding  sum(is.na(unique_ICD_codes_v15$Default_CCSR_Category.admit)) # Previous coding  unique_ICD_codes_v15 <- unique_ICD_codes_v15 %>% select(-c(Default_CCSR_Category.admit, ICD_10, Default_CCSR_Category_Desc))  # Fix column nomenclature  unique_ICD_codes_v15 <- unique_ICD_codes_v15 %>% rename(ICD_10 = ICD_10.admit)  list(unique_ICD_codes_v15$ICD_10[is.na(unique_ICD_codes_v15$Default_CCSR_Category)])  n_distinct(unique_ICD_codes_v15$Default_CCSR_Category)  # Step 4. Map back the descriptions  # Step 4.1 Create data frames of the descriptions  CCSR_desc <- CCSR %>% select(c(Default_CCSR_Category, Default_CCSR_Category_Desc))  CCSR_desc <- paste(CCSR_desc$Default_CCSR_Category, CCSR_desc$Default_CCSR_Category_Desc, sep = "_" )  CCSR_desc <- unique(CCSR_desc)  CCSR_desc <- as.data.frame(CCSR_desc)  CCSR_desc <- rename(CCSR_desc, Default_CCSR_Category_Desc = CCSR_desc)  CCSR_mod2_non_dupli_ICD_desc <- CCSR_mod2_non_dupli_ICD %>% select(c(Default_CCSR_Category, Default_CCSR_Category_Desc))  CCSR_mod2_non_dupli_ICD_desc <- paste(CCSR_mod2_non_dupli_ICD_desc$Default_CCSR_Category, CCSR_mod2_non_dupli_ICD_desc$Default_CCSR_Category_Desc, sep = "_" )  CCSR_mod2_non_dupli_ICD_desc <- unique(CCSR_mod2_non_dupli_ICD_desc)  CCSR_mod2_non_dupli_ICD_desc <- as.data.frame(CCSR_mod2_non_dupli_ICD_desc)  CCSR_mod2_non_dupli_ICD_desc <- rename(CCSR_mod2_non_dupli_ICD_desc, Default_CCSR_Category_Desc = CCSR_mod2_non_dupli_ICD_desc)  CCSR_mod3_final_desc <- CCSR_mod3_final %>% select(c(Default_CCSR_Category, Default_CCSR_Category_Desc))  CCSR_mod3_final_desc$Default_CCSR_Category <- gsub("[\\s\\p{Z}\\p{C}]+$", "", CCSR_mod3_final_desc$Default_CCSR_Category, perl = TRUE) # Remove trailing spaces and non-printable characters only  CCSR_mod3_final_desc$Default_CCSR_Category_Desc <- gsub("[\|\\s\\p{Z}\\p{C}]+$", "", CCSR_mod3_final_desc$Default_CCSR_Category_Desc, perl = TRUE) # Remove trailing '\|' and ALL non-printable characters including Unicode ones  CCSR_mod3_final_desc <- paste(CCSR_mod3_final_desc$Default_CCSR_Category, CCSR_mod3_final_desc$Default_CCSR_Category_Desc, sep = "_" )  CCSR_mod3_final_desc <- unique(CCSR_mod3_final_desc)  CCSR_mod3_final_desc <- as.data.frame(CCSR_mod3_final_desc)  CCSR_mod3_final_desc <- rename(CCSR_mod3_final_desc, Default_CCSR_Category_Desc = CCSR_mod3_final_desc)  CCSR_mod4_non_dupli_ICD_desc <- CCSR_mod4_non_dupli_ICD %>% select(c(Default_CCSR_Category, Default_CCSR_Category_Desc))  CCSR_mod4_non_dupli_ICD_desc$Default_CCSR_Category <- gsub("[\\s\\p{Z}\\p{C}]+$", "", CCSR_mod4_non_dupli_ICD_desc$Default_CCSR_Category, perl = TRUE) # Remove trailing spaces and non-printable characters only  CCSR_mod4_non_dupli_ICD_desc$Default_CCSR_Category <- trimws(CCSR_mod4_non_dupli_ICD_desc$Default_CCSR_Category, which = "right") # Remove trailing '\|' and ALL non-printable characters including Unicode ones  CCSR_mod4_non_dupli_ICD_desc$Default_CCSR_Category_Desc <- gsub("[\|\\s\\p{Z}\\p{C}]+$", "", CCSR_mod4_non_dupli_ICD_desc$Default_CCSR_Category_Desc, perl = TRUE)  CCSR_mod4_non_dupli_ICD_desc <- paste(CCSR_mod4_non_dupli_ICD_desc$Default_CCSR_Category, CCSR_mod4_non_dupli_ICD_desc$Default_CCSR_Category_Desc, sep = "_" )  CCSR_mod4_non_dupli_ICD_desc <- unique(CCSR_mod4_non_dupli_ICD_desc)  CCSR_mod4_non_dupli_ICD_desc <- as.data.frame(CCSR_mod4_non_dupli_ICD_desc)  CCSR_mod4_non_dupli_ICD_desc <- rename(CCSR_mod4_non_dupli_ICD_desc, Default_CCSR_Category_Desc = CCSR_mod4_non_dupli_ICD_desc)  CCSR_mod5_final_desc <- CCSR_mod5_final %>% select(c(Default_CCSR_Category, Default_CCSR_Category_Desc))  CCSR_mod5_final_desc$Default_CCSR_Category <- gsub("[\\s\\p{Z}\\p{C}]+$", "", CCSR_mod5_final_desc$Default_CCSR_Category, perl = TRUE) # Remove trailing spaces and non-printable characters only  CCSR_mod5_final_desc$Default_CCSR_Category <- trimws(CCSR_mod5_final_desc$Default_CCSR_Category, which = "right") # Remove trailing '\|' and ALL non-printable characters including Unicode ones  CCSR_mod5_final_desc$Default_CCSR_Category_Desc <- gsub("[\|\\s\\p{Z}\\p{C}]+$", "", CCSR_mod5_final_desc$Default_CCSR_Category_Desc, perl = TRUE)  CCSR_mod5_final_desc <- paste(CCSR_mod5_final_desc$Default_CCSR_Category, CCSR_mod5_final_desc$Default_CCSR_Category_Desc, sep = "_" )  CCSR_mod5_final_desc <- unique(CCSR_mod5_final_desc)  CCSR_mod5_final_desc <- as.data.frame(CCSR_mod5_final_desc)  CCSR_mod5_final_desc <- rename(CCSR_mod5_final_desc, Default_CCSR_Category_Desc = CCSR_mod5_final_desc)  CCSR_mod6_non_dupli_ICD_desc <- CCSR_mod6_non_dupli_ICD %>% select(c(Default_CCSR_Category, Default_CCSR_Category_Desc))  CCSR_mod6_non_dupli_ICD_desc$Default_CCSR_Category <- gsub("[\\s\\p{Z}\\p{C}]+$", "", CCSR_mod6_non_dupli_ICD_desc$Default_CCSR_Category, perl = TRUE) # Remove trailing spaces and non-printable characters only  CCSR_mod6_non_dupli_ICD_desc$Default_CCSR_Category <- trimws(CCSR_mod6_non_dupli_ICD_desc$Default_CCSR_Category, which = "right") # Remove trailing '\|' and ALL non-printable characters including Unicode ones  CCSR_mod6_non_dupli_ICD_desc$Default_CCSR_Category_Desc <- gsub("[\|\\s\\p{Z}\\p{C}]+$", "", CCSR_mod6_non_dupli_ICD_desc$Default_CCSR_Category_Desc, perl = TRUE)  CCSR_mod6_non_dupli_ICD_desc <- paste(CCSR_mod6_non_dupli_ICD_desc$Default_CCSR_Category, CCSR_mod6_non_dupli_ICD_desc$Default_CCSR_Category_Desc, sep = "_" )  CCSR_mod6_non_dupli_ICD_desc <- unique(CCSR_mod6_non_dupli_ICD_desc)  CCSR_mod6_non_dupli_ICD_desc <- as.data.frame(CCSR_mod6_non_dupli_ICD_desc)  CCSR_mod6_non_dupli_ICD_desc <- rename(CCSR_mod6_non_dupli_ICD_desc, Default_CCSR_Category_Desc = CCSR_mod6_non_dupli_ICD_desc)  CCSR_mod7_final_desc <- CCSR_mod7_final %>% select(c(Default_CCSR_Category, Default_CCSR_Category_Desc))  CCSR_mod7_final_desc$Default_CCSR_Category <- gsub("[\\s\\p{Z}\\p{C}]+$", "", CCSR_mod7_final_desc$Default_CCSR_Category, perl = TRUE) # Remove trailing spaces and non-printable characters only  CCSR_mod7_final_desc$Default_CCSR_Category <- trimws(CCSR_mod7_final_desc$Default_CCSR_Category, which = "right") # Remove trailing '\|' and ALL non-printable characters including Unicode ones  CCSR_mod7_final_desc$Default_CCSR_Category_Desc <- gsub("[\|\\s\\p{Z}\\p{C}]+$", "", CCSR_mod7_final_desc$Default_CCSR_Category_Desc, perl = TRUE)  CCSR_mod7_final_desc <- paste(CCSR_mod7_final_desc$Default_CCSR_Category, CCSR_mod7_final_desc$Default_CCSR_Category_Desc, sep = "_" )  CCSR_mod7_final_desc <- unique(CCSR_mod7_final_desc)  CCSR_mod7_final_desc <- as.data.frame(CCSR_mod7_final_desc)  CCSR_mod7_final_desc <- rename(CCSR_mod7_final_desc, Default_CCSR_Category_Desc = CCSR_mod7_final_desc)  # Step 4.2 Combine all the data frames  CCSR_desc_final <- rbind(CCSR_desc,  CCSR_mod2_non_dupli_ICD_desc,  CCSR_mod3_final_desc,  CCSR_mod4_non_dupli_ICD_desc,  CCSR_mod5_final_desc,  CCSR_mod6_non_dupli_ICD_desc,  CCSR_mod7_final_desc)  CCSR_desc_final <- unique(CCSR_desc_final) # remove duplicates  CCSR_desc_final <- strsplit(CCSR_desc_final$Default_CCSR_Category_Desc, "_") # String split  CCSR_desc_final <- data.frame(matrix(unlist(CCSR_desc_final), ncol = 2, byrow = TRUE)) # Convert the split results back to a data frame (if needed)  CCSR_desc_final <- CCSR_desc_final %>% rename(Default_CCSR_Category = X1, Default_CCSR_Category_Desc = X2) # Final table of category mapped to description  # Step 5. Final steps append and cleaning  # Step 5.1 Append descriptions  unique_ICD_codes_v16 <- unique_ICD_codes_v15  unique_ICD_codes_v16$Default_CCSR_Category <- gsub("[\\s\\p{Z}\\p{C}]+$", "", unique_ICD_codes_v16$Default_CCSR_Category, perl = TRUE)  unique_ICD_codes_v16 <- left_join(unique_ICD_codes_v16, CCSR_desc_final, by = "Default_CCSR_Category")  sum(is.na(unique_ICD_codes_v16$Default_CCSR_Category)) # number of NA CCSR categories  sum(is.na(unique_ICD_codes_v16$Default_CCSR_Category_Desc)) # number of NA CCSR descriptions  # Step 5.2 Cleaning  unique_ICD_codes_v16 <- unique_ICD_codes_v16 %>%  select(ICD_10, DIAGNOSIS_DESC, Default_CCSR_Category, Default_CCSR_Category_Desc)  View(unique_ICD_codes_v16) # View final dataset with mapped ICD-10-AM codes to CCSR categories |
